# Spatiotemporal Immune-Fibroblast Crosstalk in Pulmonary Fibrosis: From Failed Repair to Fibrotic Niche Formation

**DOI:** 10.7150/ijbs.137072

**Published:** 2026-09-24

**Authors:** Simeng Lu, Weijia Zhang, Xiaoming Zhao, Chao Feng, Wangwang Chen, Yufang Shi, Jiankai Fang

**Affiliations:** 1 The Third Affiliated Hospital of Soochow University, State Key Laboratory of Radiation Medicine and Protection, Jiangsu Key Laboratory of Radiation Injury Prevention and Hazardous Substance Control, Biomedical Basic Research Center of Jiangsu, Institutes for Translational Medicine, Suzhou Medical College of Soochow University, Suzhou, Jiangsu 215123, China.; 2 Department of Experimental Medicine, TOR, University of Rome “Tor Vergata”, Rome 00133, Italy.; 3 Laboratory Animal Center, Suzhou Medical College of Soochow University, Suzhou, Jiangsu 215123, China.; 4 Shanghai Institute of Nutrition and Health, Chinese Academy of Sciences, Shanghai 200031, China.; 5 MOE Key Laboratory of Geriatric Diseases and Immunology, Suzhou Medical College of Soochow University, Suzhou, Jiangsu 215123, China.

**Keywords:** pulmonary fibrosis, immune-fibroblast crosstalk, fibroblast heterogeneity, spatial niche, single-cell transcriptomics, spatial transcriptomics

## Abstract

Pulmonary fibrosis is a common and often fatal outcome of fibrosing interstitial lung diseases, characterized by progressive extracellular matrix deposition, destruction of alveolar architecture and irreversible loss of respiratory function. Although fibroblast activation and epithelial dysfunction are central to disease pathogenesis, fibrosis cannot be adequately explained by mesenchymal dysregulation alone. Rather, it represents a failure of multicellular repair regulation, in which immune cells, fibroblasts and epithelial populations reciprocally reshape cellular states, tissue niches and disease trajectories. Early innate immune responses amplify epithelial injury while simultaneously initiating provisional repair; however, when injury persists, these transient programmes fail to resolve and become stabilized into chronic profibrotic circuits involving monocyte-derived macrophages, dysfunctional T cells, inflammatory fibroblasts and matrix-producing myofibroblasts. Recent single-cell, spatial transcriptomic and lineage-tracing studies have revealed discrete cellular states, including SPP1⁺ scar-associated macrophages, inflammatory fibroblasts, CTHRC1⁺ myofibroblasts and tertiary-lymphoid-structure-associated stromal cells, that assemble multicellular, stage-specific and microanatomically restricted fibrotic niches. Collectively, these findings support a model in which soluble mediators, extracellular matrix mechanics, metabolic rewiring and immune-regulatory signalling cooperate to establish self-reinforcing stromal-immune circuits. In this Review, we synthesize current evidence for bidirectional immune-fibroblast crosstalk in pulmonary fibrosis, focusing on macrophage and T cell programmes, fibroblast-mediated immune regulation, spatiotemporal niche remodelling, mechanistic signalling networks, experimental models and therapeutic opportunities. We propose that progressive fibrosis reflects the spatiotemporal locking of pathological epithelial-immune-stromal circuits, a framework that may explain the limited efficacy of broad anti-inflammatory therapies in idiopathic pulmonary fibrosis and guide the development of more precise, stage-specific and combinatorial antifibrotic strategies.

## Introduction

Pulmonary fibrosis represents a final common pathway shared by diverse fibrosing interstitial lung diseases (fILDs), characterized by excessive extracellular matrix (ECM) deposition, collapse of alveolar units, traction bronchiectasis, honeycombing and, ultimately, progressive respiratory failure 1, 2. Idiopathic pulmonary fibrosis (IPF) remains the archetypal and most intensively studied subtype, but progressive fibrosis also occurs in connective-tissue-disease-associated interstitial lung disease, hypersensitivity pneumonitis and other forms of fILD 3, 4. Despite major advances in molecular profiling and clinical management, pulmonary fibrosis continues to confer a poor prognosis, and currently available antifibrotic agents slow, but do not halt or reverse, disease progression 5, 6. The recent expansion of the therapeutic armamentarium to include nerandomilast in some jurisdictions illustrates meaningful clinical progress 7, 8, but also underscores the persistent need for therapies that more effectively intercept the biological circuits sustaining fibrosis.

Historically, pulmonary fibrosis was conceptualized largely in terms of epithelial injury and fibroblast activation as parallel pathogenic processes. Over the past few years, however, this framework has shifted substantially. Fibroblasts are no longer viewed as passive structural cells whose principal function is ECM production; instead, they are increasingly recognized as immunologically active stromal cells that sense tissue injury, recruit and instruct immune cells, organize reparative niches and, under pathological conditions, lock tissues into chronic fibrotic remodelling 9, 10. Conversely, immune cells are not merely inflammatory bystanders. They actively shape fibroblast fate, determine whether wound-healing programmes resolve or persist, and influence the composition, stiffness and inflammatory tone of the fibrotic microenvironment 11, 12.

This reciprocal relationship is neither static nor spatially uniform, but evolves across both time and anatomical space. Temporally, the cellular networks that dominate early inflammatory injury differ from those that sustain established fibrosis. Spatially, fibrotic remodelling is organized into discrete microanatomical niches in which immune cells, aberrant epithelial cells, endothelial cells and fibroblast subsets communicate through highly localized signalling networks. Advances in single-cell RNA sequencing, spatial transcriptomics, imaging mass cytometry and lineage tracing have revealed discrete stromal and immune states within the fibrotic lung, including inflammatory fibroblasts, PDGFRα^+^ alveolar fibroblasts, CTHRC1^+^ myofibroblasts, SPP1^+^ macrophages, dysfunctional or exhausted T cells and tertiary-lymphoid-structure-associated fibroblasts. Importantly, the abundance, localization and interactions of these states vary across disease stages and anatomical regions 13-16.

Although several recent reviews have provided important insights into fibroblast immunoregulatory functions, immune complexity in fibrotic interstitial lung disease and the lineage evolution of injury-associated fibroblasts, an integrated framework linking cellular heterogeneity to spatial organization, temporal progression and therapeutic timing remains underdeveloped 10, 12, 17, 18. In this Review, we therefore examine pulmonary fibrosis through the lens of dynamic stromal-immune crosstalk. We address two interrelated questions: first, how do distinct immune-cell states instruct fibroblast activation, persistence and pathogenic specialization; and second, how do fibroblasts, in turn, recruit, polarize, restrain or exclude immune cells, thereby reshaping the inflammatory set point of the lung? We argue that a spatiotemporal perspective is essential for understanding how initially reparative interactions evolve into self-sustaining fibrotic circuits and for identifying therapeutic windows that remain obscured by static or reductionist models (Figure 1).

### Conceptual positioning of this review

Recent high-profile reviews have substantially advanced our understanding of immune and stromal mechanisms in pulmonary fibrosis. Collectively, these studies have established fibroblasts as active regulators of immune responses, revealed the diversity of innate immune states across fibrotic interstitial lung diseases and delineated lineage trajectories of injury-associated fibroblast populations 10, 12, 17, 18. However, these advances have generally emphasized individual cellular compartments, molecular programmes or lineage relationships, rather than the dynamic organization of reciprocal immune-stromal interactions across space and time.

Here, we propose a complementary framework in which pulmonary fibrosis is viewed as a progressive transition from transient, repair-associated cellular interactions to spatially organized and self-reinforcing fibrotic niches. This framework is built around three interconnected principles. First, the spatial organization of immune-stromal interactions determines functional niche identity. Second, reciprocal signalling among immune cells, fibroblasts and the ECM stabilizes pathological cell states and promotes their persistence. Third, therapeutic efficacy is likely to depend on disease stage, niche maturity and rational combination strategies rather than on inhibition of individual pathways in isolation.

A comparison of the major conceptual frameworks is summarized in Table 1.

## Immune-cell programmes that instruct fibroblast activation

### Macrophages: from injury response to fibrogenic niche formation

Macrophages are among the most influential immune regulators of fibroblast behaviour in pulmonary fibrosis. Although the classical M1/M2 polarization paradigm remains a useful heuristic, it does not capture the full spectrum of macrophage states now identified in fibrotic lungs. Current evidence indicates that macrophage function is shaped by lineage origin, anatomical location, stage of injury and local niche cues 19, 20. In particular, monocyte-derived macrophages appear to exert a more prominent profibrotic influence than embryonically derived resident alveolar macrophages, especially during persistent or progressive disease 21.

During early injury, recruited monocytes differentiate into inflammatory macrophages that amplify epithelial damage through IL-1β, TNF and other mediators. When tightly controlled, these responses can be protective by containing tissue injury and initiating repair. However, when epithelial damage persists, inflammatory programmes fail to resolve and macrophages transition towards states that favour fibrogenesis rather than restitution. In this context, macrophages produce TGF-β, PDGF, amphiregulin, IL-13 and other profibrotic mediators that promote fibroblast proliferation, migration and myofibroblast differentiation 22-24. Recent single-cell studies have identified SPP1^+^ macrophage states in human IPF lungs and in fibrotic tissues from other organs, suggesting that this programme represents a conserved fibrosis-associated myeloid state 25-28. Importantly, however, the evidence in human tissues remains largely associative, whereas direct functional support for SPP1^+^ macrophages in promoting fibroblast activation and sustaining fibrotic niches derives predominantly from experimental fibrosis models and mechanistic studies. Single-cell and spatial profiling of human and experimental lung and liver fibrosis further show that these macrophages frequently co-express scar-associated markers, including *TREM2* and *CD9*, and localize adjacent to fibroblastic foci and activated mesenchymal cells 29. This spatial coupling supports the idea that SPP1^+^ macrophages are not merely a consequence of fibrosis, but active components of the fibrogenic niche 30, 31. Among the candidate pathways linking these macrophages to fibroblast activation, SPP1-CD44 signalling has emerged as a particularly plausible axis, with TGF-β, PDGF and CSF1 signalling providing additional reinforcing inputs 32, 33.

Macrophages also promote fibrosis indirectly by reshaping the epithelial context in which fibroblasts operate. Chronic macrophage-derived IL-1β impairs effective alveolar regeneration and promotes dysplastic repair 34. In parallel, macrophage-derived mediators can stabilize aberrant transitional epithelial states, thereby sustaining epithelial stress signals that perpetuate fibroblast activation 35. Macrophage-fibroblast crosstalk is therefore inseparable from macrophage-epithelial crosstalk, supporting a model in which pulmonary fibrosis emerges from interconnected multicellular circuits rather than from isolated binary interactions.

A central example of such circuitry is the self-reinforcing macrophage-fibroblast-matrix loop. Macrophage-derived mediators, including TGF-β, PDGF and SPP1, promote the acquisition and persistence of matrix-producing fibroblast and myofibroblast states, accompanied by sustained expression of ECM-associated genes such as *COL1A1*, *POSTN* and *FN1* and by enhanced matrix deposition and remodelling 32, 36, 37. These fibroblast states are further supported by metabolic reprogramming towards anabolic pathways, including glycolysis and glutamine metabolism, that sustain the biosynthetic demands of ECM production 38-40. In turn, persistently activated fibroblasts alter their secretory programmes, producing mediators such as TGF-β, CSF1 and CXCL12 while continuing to deposit and remodel ECM 41-43. Progressive matrix accumulation and stiffening then amplify mechanotransductive signalling, including integrin-dependent activation of latent TGF-β, and may favour the persistence of profibrotic macrophage states characterized by high SPP1, TREM2 and CD9 expression together with altered lipid-metabolic programmes 44-48. Thus, macrophage activation, fibroblast-state reprogramming and matrix remodelling form a mutually reinforcing circuit in which each component stabilizes the others, converting otherwise transient repair-associated interactions into a persistent fibrogenic niche.

### T cells and adaptive immunity: from helper polarization to exhaustion

Adaptive immunity contributes to pulmonary fibrosis in a far more heterogeneous and stage-dependent manner than was once appreciated. Earlier models emphasized a Th1-Th2 balance, in which Th1 cytokines such as IFN-γ were considered broadly protective, whereas Th2 cytokines such as IL-4 and IL-13 were considered profibrotic 49-52. Although this framework remains informative, particularly because IL-4 and IL-13 can promote fibroblast activation both directly and indirectly through macrophage polarization, it no longer captures the full complexity of adaptive immunity in fibrosis. Th17 responses, regulatory T cell plasticity, cytotoxic T cell dysfunction, B cell activation and tertiary lymphoid structure formation all contribute to the adaptive immune landscape of fibrotic lungs 53-57. Importantly, the relative contribution of these programmes changes over the course of disease. Th1-associated responses are most prominent during early tissue injury and are generally linked to inflammatory control, tissue repair and antifibrotic activity. As repair fails and chronic inflammation becomes established, Th2- and Th17-associated programmes increasingly favour fibroblast activation and ECM deposition. In established fibrosis, T cell dysfunction, sustained B cell activation and tertiary lymphoid structures contribute to the persistence of a pathological immune microenvironment. Regulatory T cells represent an important exception to this simple temporal progression, as their effects can be either protective or profibrotic depending on the local microenvironment and stage of disease.

Within this broader temporal framework, distinct T cell programmes exert divergent effects on fibroblast behaviour. Th1-associated responses can restrain fibrosis by antagonizing TGF-β signalling and limiting collagen deposition 58. By contrast, Th2 cells enhance fibroblast proliferation and matrix synthesis through IL-4 and IL-13 59, 60. Th17 cells add a further pathogenic dimension through IL-17A, which can promote fibroblast proliferation, augment neutrophil recruitment, amplify local inflammation and cooperate with TGF-β-dependent profibrotic pathways 53, 61, 62. In some experimental contexts, PD-1^+^ Th17 cells have also been identified as an important source of TGF-β, providing a more direct link between maladaptive T cell states and fibroblast activation 63.

T cell dysfunction may become particularly relevant as fibrosis becomes established. T cells in fibrotic lungs frequently upregulate inhibitory receptors such as PD-1, TIM-3 and CTLA-4 and display impaired proliferative and cytotoxic capacity 64, 65. Such dysfunctional or exhaustion-like states may facilitate fibrotic persistence by weakening immune surveillance of senescent or aberrantly activated stromal cells, while residual production of profibrotic mediators, including IL-17A and TGF-β1, may further reinforce stromal activation 66. However, direct evidence for this mechanism in human pulmonary fibrosis remains limited, and much of the functional support derives from experimental models and mechanistic studies. Conversely, the fibrotic stroma itself can promote T cell dysfunction 65, 67, indicating that adaptive immune impairment is both a potential driver and a consequence of chronic fibrotic niche formation.

This reciprocal coupling establishes a self-reinforcing T cell-fibroblast circuit. Activated fibroblasts produce cytokines and chemokines, including IL-6, IL-1β, TGF-β and selected chemotactic factors, that can promote Th17 differentiation, perturb regulatory T cell function and favour dysfunctional T cell states. T cells, in turn, acquire exhaustion-associated transcriptional programmes characterized by sustained inhibitory-receptor expression and metabolic adaptations that constrain proliferative and cytotoxic capacity 64, 65, 68. Reduced immune surveillance can permit pathological stromal cells to persist, whereas continued production of IL-17A and TGF-β can promote fibroblast proliferation, collagen synthesis and maintenance of myofibroblast phenotypes 53, 63, 69. Thus, fibroblasts do not simply respond to adaptive immune signals; they actively shape local T cell states, which in turn feed back to reinforce fibroblast activation and pathological matrix remodelling.

B cells and tertiary lymphoid structures add a further layer of adaptive immune remodelling. Under conditions of chronic inflammation, fibroblasts can acquire lymphoid-organizer-like functions, including expression of CCL19, CCL21 and CXCL13, thereby promoting the assembly and maintenance of tertiary lymphoid structures 70. In pulmonary fibrosis, these tertiary-lymphoid-structure-associated stromal niches may sustain local lymphocyte activation, autoantibody production and chronic inflammation 71, 72. Such processes are likely to be particularly relevant in autoimmune-associated interstitial lung disease, but may also contribute to subsets of IPF characterized by pronounced immune remodelling (Figure 2). B cells and tertiary lymphoid structures are therefore more plausibly linked to the maintenance and compartmentalization of chronic adaptive immune responses than to the initial induction of pulmonary fibrosis, although their contribution is likely to vary across disease subtype, immune phenotype and stage.

Beyond conventional adaptive lymphocytes, dendritic cells (DCs), natural killer (NK) cells and group 2 innate lymphoid cells (ILC2s) further diversify the immune-stromal landscape of pulmonary fibrosis 73. Conventional type 1 and type 2 DCs (cDC1s and cDC2s), together with monocyte-derived DCs (mo-DCs), shape local adaptive immunity through antigen presentation, T cell priming and helper T cell polarization and may thereby influence fibroblast activation indirectly through cytokine-network remodelling 74, 75. ILC2s provide an additional profibrotic input through the production of IL-5, IL-13 and amphiregulin, reinforcing type 2 immunity and macrophage-fibroblast crosstalk and favouring the establishment of local profibrotic niches 76-79. By contrast, NK cells may exert a protective function by limiting the accumulation of senescent or persistently activated fibroblasts through cytotoxic immune surveillance. Experimental studies indicate that activated NK cells can recognize and eliminate senescent lung fibroblasts through granule-mediated cytotoxicity and death-receptor-dependent mechanisms, thereby attenuating, and in some settings reversing, experimental pulmonary fibrosis 80. Although the contributions of these populations remain less well defined than those of macrophages and conventional adaptive lymphocytes, they broaden the cellular repertoire through which immune cells regulate fibroblast states and fibrotic niche dynamics.

## Fibroblasts as active regulators of immunity

### Inflammatory fibroblasts and immune-cell recruitment

Fibroblasts are increasingly recognized as integral components of the extended innate immune system 81, 82. In the healthy lung, distinct fibroblast subsets maintain alveolar architecture, support epithelial stemness, regulate ECM turnover and preserve tissue homeostasis. Following injury, however, fibroblasts rapidly reprogramme their transcriptional states and adopt inflammatory, proliferative or myofibroblastic phenotypes in response to local cues. Importantly, these state transitions are not simply downstream consequences of inflammation; they actively reshape the local immune environment by regulating immune-cell recruitment, retention and functional differentiation 83.

A central feature of this immunoregulatory capacity is the ability of fibroblasts to sense tissue perturbation and translate it into inflammatory signalling. Activated fibroblasts respond to pathogen-associated and damage-associated molecular patterns by producing chemokines, cytokines, adhesion molecules and acute-phase mediators 84, 85. In experimental lung injury, alveolar fibroblasts can transition into inflammatory states characterized by expression of genes such as *Ccl2*, *Cxcl2*, *Saa3* and *Lcn2* 17. Through these programmes, fibroblasts are positioned to recruit CCR2^+^ monocytes, neutrophils and other myeloid populations into damaged tissue. As inflammation becomes chronic, additional fibroblast-derived chemokine axes, including CXCL12-CXCR4 and CXCL13-CXCR5, can promote lymphocyte recruitment, retention and spatial organization, thereby contributing to the establishment of tertiary lymphoid structures 86.

Beyond controlling immune-cell trafficking, fibroblasts can also shape the functional states of recruited immune cells. Activated fibroblasts produce TGF-β and CSF1 and can also reshape the local metabolic and lipid-associated microenvironment in ways that support the differentiation or persistence of profibrotic macrophage states 41, 42, 87. Within the adaptive immune compartment, fibroblast-derived cytokines, including IL-6 and TGF-β, together with locally available IL-1β, can favour Th17 differentiation and persistence 68. Fibroblasts therefore do more than recruit immune cells into fibrotic lesions: they help determine which immune-cell states are established and maintained within the local niche, thereby converting inflammatory recruitment into sustained immune-stromal coupling.

This immunoregulatory capacity is itself heterogeneous across fibroblast states. Fibroblasts within the fibrotic lung are not functionally equivalent: some populations support epithelial repair and barrier integrity, whereas others specialize in inflammatory signalling, immune-cell recruitment and retention, or pathological matrix production 88, 89. Fibroblast heterogeneity is therefore not merely transcriptional but functionally consequential for the organization of local immunity. Accordingly, “the fibroblast” can no longer be regarded as a single therapeutic entity. Effective interventions will need to distinguish pathogenic inflammatory and matrix-producing states from fibroblast populations that maintain alveolar homeostasis or support regeneration, thereby disrupting disease-promoting immune-stromal circuits without compromising physiological repair.

### Myofibroblasts, immune suppression and failed clearance

In advanced fibrosis, fibroblast activation culminates in the persistence of contractile, ECM-producing myofibroblast states. These cells have traditionally been regarded primarily as effectors of matrix deposition, but accumulating evidence indicates that they can also modulate local immune responses in ways that favour their persistence. This expanded view recasts myofibroblasts not simply as matrix-producing cells, but as active components of self-reinforcing pathological niches 10. In end-stage IPF, subsets of activated fibroblasts and myofibroblasts exhibit increased expression of immune-regulatory molecules such as PD-L1 and CD47, which can attenuate T cell activity through PD-1 signalling and restrict macrophage-mediated phagocytosis, respectively 64, 66, 67. Together, these programmes may create a locally immunosuppressive environment in which pathological stromal cells become less susceptible to immune-mediated clearance. TGF-β-rich stromal signalling can further restrict cytotoxic T cell access and function within fibrotic lesions, thereby weakening local immune surveillance 90. Impaired clearance may, in turn, favour the persistence of myofibroblast states with enhanced immune-evasive properties, establishing a positive-feedback circuit in which stromal immune suppression promotes myofibroblast survival and persistent myofibroblasts further consolidate the immunosuppressive niche.

Failure of fibroblast clearance has important consequences for fibrosis resolution. During successful tissue repair, activated fibroblasts can be removed or deactivated through apoptosis, dedifferentiation, senescence-associated state transitions or immune-mediated clearance. In progressive fibrosis, these resolution programmes become incomplete or dysregulated, allowing pathological myofibroblast states to persist and ECM production to continue 91, 92. Dysfunctional adaptive immune responses may further exacerbate this process by reducing the cytotoxic surveillance required to eliminate senescent or aberrantly activated stromal cells 93, 94. Importantly, senescence itself is context dependent: transient senescence can contribute to resolution, whereas persistent senescent stromal populations may reinforce chronic inflammation and fibrotic remodelling through altered secretory programmes. Thus, failed fibrosis resolution reflects not simply continued fibroblast activation, but defective termination of pathological stromal states.

Related observations in other chronic pathological tissues support the broader relevance of this principle. In tumours, LRRC15^+^ myofibroblastic cancer-associated fibroblasts are associated with suppression of CD8^+^ T cell effector responses, whereas stromal TGF-β signalling contributes to immune exclusion 90, 95. Although the cellular context and selective pressures in cancer differ fundamentally from those in non-neoplastic fibrosis, these parallels raise the possibility that immune evasion is a recurring property of chronically activated fibroblastic niches. Pulmonary fibrosis may therefore share selected organizational features with other immune-excluded pathological microenvironments, particularly the reciprocal coupling of stromal persistence and impaired immune surveillance (Figure 3).

More broadly, these interactions illustrate that immune-stromal crosstalk in pulmonary fibrosis is not organized as a collection of independent, unidirectional signalling axes, but as interconnected feedback circuits operating across spatial and temporal scales 12, 17. Macrophage-fibroblast interactions promote ECM deposition and alter tissue mechanics; increased matrix stiffness and mechanotransduction then reinforce profibrotic fibroblast programmes and can further influence macrophage states. In parallel, aberrant epithelial cells and dysfunctional T cells provide additional signals that reshape macrophage, fibroblast and lymphocyte phenotypes 37, 96, 97. Local cell-state transitions can therefore propagate through neighbouring cellular compartments, progressively converting initially discrete interactions into a coordinated and self-maintaining fibrotic network.

This systems-level model also clarifies the distinction between observations in human disease and mechanistic inference. Studies of human IPF provide strong evidence for the coexistence and spatial organization of disease-associated epithelial, immune and stromal states, but they generally do not establish the temporal ordering or causal direction of the feedback circuits linking these populations. Much of the evidence supporting causality derives instead from experimental fibrosis models, perturbation studies and mechanistic systems. Taken together, these data support the possibility that progressive integration of immune, stromal, epithelial and matrix feedback represents a functional tipping point at which reparative plasticity is lost and tissue remodelling becomes self-sustaining. This framework further suggests that targeting a single cell population or mediator may be insufficient once fibrosis is established; therapeutic strategies that disrupt the coupling between multiple pathological circuits may be more effective in destabilizing the fibrotic niche and restoring productive tissue repair.

## Spatiotemporal remodelling of the fibrotic niche

### Temporal programmes underlying fibrotic niche formation

The transition from acute injury to chronic fibrosis remains one of the central unresolved questions in pulmonary fibrosis. Early injury responses are not inherently pathological; rather, many are essential for epithelial protection, debris clearance and wound closure. The critical defect arises when these programmes fail to terminate appropriately. Under persistent or repetitive injury, normally transient reparative states become prolonged, and pathological cell states progressively stabilize through reciprocal interactions with neighbouring cells and the ECM 98. As these states become increasingly coupled through interdependent feedback circuits, tissue repair loses plasticity and shifts towards a self-sustaining pathological programme. This progressive loss of reversibility provides the temporal foundation for the emergence and subsequent stabilization of fibrotic niches.

#### Acute injury initiates physiological repair programmes

During acute lung injury, neutrophils, inflammatory monocytes, dendritic cells and early macrophage states coordinate the initial inflammatory response. Neutrophils release reactive oxygen species, elastase, matrix metalloproteinases and neutrophil extracellular traps (NETs), which contribute to pathogen and debris clearance but, when excessive or persistent, can exacerbate epithelial injury 99-102. NETs may further promote fibrosis by carrying mediators with profibrotic activity and amplifying macrophage activation 103. In parallel, recruited myeloid cells begin to produce provisional repair signals, including TGF-β, thereby initiating fibroblast activation and matrix remodelling before a chronic fibrotic programme has been established 104. Thus, the earliest stromal responses arise within a physiological repair programme, but already contain signalling components that can become pathogenic if their duration or magnitude is not appropriately constrained.

Whether this acute response resolves or progresses towards fibrosis depends on the temporal coordination of inflammatory resolution, epithelial regeneration and termination of stromal repair programmes. Following effective removal of the injurious stimulus, inflammatory and damage-associated signals decline, and macrophage populations adopt repair-associated functions that facilitate debris clearance, inflammation resolution and matrix remodelling through mediators such as IL-10, growth factors and pro-phagocytic programmes 105-108. Concurrently, alveolar type 2 epithelial (AT2) cells proliferate and differentiate towards alveolar type 1 epithelial (AT1) cells under the control of regenerative signalling networks that include WNT, FGF and YAP/TAZ pathways, thereby contributing to restoration of alveolar barrier integrity and tissue homeostasis 109-112. Fibroblast activation must likewise contract as repair proceeds, allowing reparative stromal states to return towards quiescence or undergo clearance.

By contrast, persistent injury or defective resolution disrupts this coordinated sequence. Continued epithelial stress, sustained inflammatory signalling and incomplete termination of fibroblast activation progressively divert the tissue away from productive regeneration and towards maladaptive repair. Importantly, this transition does not represent an abrupt switch between discrete acute and chronic phases. Rather, it comprises a continuum of partially overlapping tissue states encompassing inflammatory activation, provisional repair, divergent cell-fate decisions, failed regeneration and progressive pathological stabilization. Along this trajectory, cellular identities, intercellular signalling networks and the surrounding ECM are continuously remodelled, gradually reducing the capacity of the tissue to return to homeostasis. Fibrosis therefore emerges when reparative plasticity is progressively lost and transient injury-response programmes become incorporated into persistent, self-reinforcing cellular and matrix networks.

#### Crossing the repair threshold: molecular determinants of failed repair

Whether severe acute lung injury (ALI), acute respiratory distress syndrome (ARDS) or other forms of persistent lung injury ultimately resolve or progress towards fibrosis depends on the temporal coordination of inflammatory resolution, epithelial regeneration and termination of stromal repair programmes. When severe or persistent injury prevents the timely attenuation of inflammatory and profibrotic signalling, otherwise reparative pathways remain active beyond the window required for effective regeneration and progressively redirect tissue towards maladaptive remodelling 113-116. This transition is unlikely to be governed by the transient elevation of any single mediator. Rather, it reflects the integrated effects of signal duration, magnitude and combination — including context-dependent inputs from TGF-β, IL-1β, TNF and IFN-γ — together with the regenerative capacity and mechanical state of the surrounding tissue 52, 117. Accordingly, no universal molecular concentration threshold has been established across ALI or ARDS models. Here, the 'repair threshold' should instead be understood as a functional threshold at which cumulative inflammatory, epithelial, stromal and mechanical perturbations exceed the capacity of tissue homeostatic programmes to restore a reversible state.

Persistent inflammatory signalling is one mechanism that drives this loss of reversibility. Sustained STAT1- and NF-κB-dependent transcriptional programmes, coupled to HIF-1α-mediated metabolic reprogramming and IL-1β production, can prolong inflammatory myeloid states 118-121. Within this environment, recruited monocytes follow altered differentiation trajectories towards disease-associated alveolar macrophage states that acquire profibrotic transcriptional features and fail to fully establish homeostatic or inflammation-resolving programmes 24, 122. Once stabilized, these macrophage states produce TGF-β, IL-1β, S100A4 and multiple chemokines that reinforce monocyte recruitment, fibroblast activation and ECM deposition 123-125. Fibroblasts exposed to these persistent signals correspondingly shift from transient repair-associated states towards matrix-producing and myofibroblast programmes. Thus, failed resolution of the myeloid response is better understood as progressive stabilization of pathological macrophage-fibroblast signalling than as defective 'repolarization' driven by a single aberrant cytokine.

In parallel, persistent epithelial stress progressively compromises the regenerative compartment. DNA damage, oxidative stress and telomere dysfunction activate p53-p21-dependent stress and senescence programmes in AT2 cells and perturb regenerative and mechanosensitive pathways, including WNT and YAP/TAZ signalling, thereby reducing proliferative and differentiation capacity 112, 126-128. Importantly, failed regeneration does not simply reflect depletion of AT2 progenitors. It also involves accumulation of epithelial cells arrested in aberrant transitional states, including KRT8^+^ alveolar differentiation intermediates. Under physiological conditions, such states are transient intermediates during AT2-to-AT1 differentiation; under persistent injury, sustained IL-1β, IL-11 and other stress-associated signals can prevent their resolution and impair maturation towards functional AT1 cells 35, 129-131. These persistent transitional epithelial populations can, in turn, maintain a profibrotic microenvironment through continued production of cytokines, chemokines and other injury-associated signals, thereby coupling defective epithelial regeneration to sustained stromal and immune activation.

The influence of aberrant epithelial states extends beyond failed regeneration to active remodelling of the local immune niche. During acute injury, damage-associated molecular patterns (DAMPs) and alarmins transiently activate innate immunity and normally decline as tissue integrity is restored. When epithelial injury persists, however, signals such as HMGB1, extracellular ATP and IL-33 can remain elevated within the alveolar microenvironment 129, 132. Through pattern-recognition receptors, purinergic signalling and the IL-33-ST2 axis, these mediators sustain recruitment and activation of monocyte-macrophage populations and other innate immune compartments 77, 132, 133. Dysregulated WNT activity may further alter epithelial regenerative and inflammatory programmes, while changes in the lipid-rich fibrotic microenvironment — including sphingolipids, phospholipids and other candidate TREM2 ligands — can engage TREM2-associated survival and metabolic programmes in monocyte-derived macrophages 134-137. Together, these epithelial-immune interactions shift the tissue response from transient danger sensing towards persistent immune reprogramming, establishing conditions that favour subsequent immune-fibroblast coupling.

Persistent pathological states can then become progressively stabilized at the epigenetic and mechanical levels. Single-cell multi-omic and epigenomic studies indicate that alterations in DNA methylation, chromatin accessibility and histone modifications are not merely consequences of fibrosis, but can contribute to the maintenance of disease-associated transcriptional programmes 138-140. Chronic inflammatory signalling, TGF-β activity and mechanical stress remodel regulatory landscapes associated with inflammatory and profibrotic genes and reinforce transcriptional networks involving AP-1, SMAD and YAP/TAZ-TEAD 140-142. These programmes can reduce the capacity of aberrant epithelial cells, disease-associated macrophages and activated fibroblasts to return towards homeostatic states, even when the initiating injury is partially attenuated. Epigenetic memory, paracrine signalling and ECM-derived mechanical feedback can therefore act together to stabilize cellular states that were initially adaptive and transient.

The transition across the repair threshold should consequently be viewed as a continuum of partially overlapping functional checkpoints rather than as a single discrete event. Early injury is dominated by neutrophil recruitment and inflammatory monocyte-macrophage responses; successful repair subsequently requires inflammatory resolution, macrophage state normalization, AT2-driven alveolar regeneration, fibroblast deactivation and matrix remodelling 12. When these restorative programmes remain coordinated, the tissue retains sufficient plasticity to return towards homeostasis. When they progressively diverge, however, AT2 cells remain trapped in abnormal transitional states, monocyte-derived macrophages retain disease-associated programmes, fibroblasts persist in matrix-producing states and the ECM becomes increasingly stiff and crosslinked 129, 130. These changes mutually reinforce one another, causing transient repair programmes to become incorporated into stable pathological networks.

Thus, the key distinction between reversible repair and persistent fibrosis is not the absolute magnitude of any individual pathological signal or the appearance of a single cellular marker, but whether coordinated recovery programmes can still be re-established. A reversible repair niche retains the capacity for inflammatory resolution, epithelial maturation, normalization of macrophage states, fibroblast deactivation and ECM turnover. By contrast, a fibrotic niche emerges when these restorative trajectories lose reversibility and become constrained by persistent inflammatory signalling, aberrant epithelial differentiation, stabilized immune and fibroblast states, epigenetic memory and pathological matrix mechanics. From this perspective, crossing the repair threshold represents a progressive loss of tissue plasticity that provides the temporal basis for subsequent fibrotic niche formation.

#### Loss of tissue plasticity establishes the foundation for fibrotic niche formation

When injury resolves, inflammatory programmes contract, immune-cell numbers decline and activated fibroblasts either return towards quiescence or are eliminated 143. When epithelial regeneration remains incomplete, however, these resolution trajectories progressively diverge. Monocyte-derived macrophages accumulate and can replace or supplement resident alveolar macrophage populations, T cell states become increasingly dysfunctional, and fibroblasts shift from transient inflammatory or niche-supportive programmes towards persistent matrix-producing and immune-regulatory states 64, 122, 144. Together, these changes promote sustained myofibroblast activity, progressive ECM accumulation and stiffening, and increasing distortion of alveolar architecture.

Failed repair does not progress through a simple linear sequence. Rather, loss of tissue plasticity emerges as pathological cell states become coupled through mutually reinforcing feedback circuits. Profibrotic macrophages provide TGF-β and other fibrogenic signals that sustain fibroblast activation and ECM deposition. The resulting increase in matrix stiffness enhances integrin-dependent mechanotransduction and promotes activation of matrix-sequestered latent TGF-β, thereby further stabilizing profibrotic fibroblast programmes 44-46. In parallel, aberrant transitional epithelial cells maintain chemokine and injury-associated signalling that recruits and activates immune populations and perpetuates tissue-remodelling responses 35, 129. These processes increasingly couple epithelial dysfunction, immune reprogramming, fibroblast activation and matrix mechanics into an integrated pathological network.

As these feedback circuits strengthen, tissue remodelling becomes progressively less dependent on the original injurious stimulus and increasingly sustained by local interactions among disease-associated cell states and the remodelled ECM. Importantly, this transition should not be interpreted as an abrupt acquisition of complete injury independence. Instead, it represents a progressive shift from an injury-responsive and reversible repair programme towards a locally self-sustaining state that is increasingly resistant to resolution. Temporal persistence of pathological cell states therefore provides one prerequisite for fibrotic niche formation, but persistence alone does not fully explain how these states become stabilized within tissue.

A second requirement is spatial organization. Pathological epithelial states, disease-associated immune populations, activated fibroblasts and remodelled ECM must become locally coordinated within specific microanatomical environments in which short-range signalling, cell-cell contact and matrix-derived cues can be repeatedly reinforced. We refer to the convergence of persistent pathological cell states with stable spatial organization as spatiotemporal locking: a state in which cellular trajectories and local niche architecture mutually constrain one another, reducing the capacity of the tissue to return towards homeostasis. In this framework, temporal persistence creates the substrate for niche formation, whereas spatial organization consolidates and localizes the pathological feedback circuits that maintain it. Defining how these fibrotic niches acquire, expand and stabilize their spatial architecture is therefore essential for understanding the transition from failed repair to persistent fibrosis.

### Spatial organization of the fibrotic niche

The spatial principles that govern fibrotic niche formation remain incompletely understood. Persistent pathological cell states alone are unlikely to account for progressive tissue remodelling; these states must also become organized within defined microanatomical regions where short-range signalling, cell-cell interactions and matrix-derived cues can be repeatedly reinforced. A central question is therefore how disease-associated epithelial, immune and stromal states reorganize the normal cellular architecture of the lung to generate pathological spatial units with characteristic cellular compositions, molecular programmes and functional interdependencies. Advances in single-cell sequencing and spatial omics now enable these disease-associated states to be resolved within their native tissue context, revealing how cellular identity is coupled to anatomical localization and local neighbourhood composition. Together, these approaches provide a framework for defining how fibrotic niches emerge, expand and become spatially stabilized during disease progression 17, 30, 31, 145.

#### Single-cell and spatial omics define the landscape of fibrotic microenvironments

Single-cell and spatial omics technologies have fundamentally reshaped our understanding of pulmonary fibrosis by revealing that the fibrotic lung is not a homogeneous pathological entity, but a mosaic of spatially distinct yet functionally interconnected microenvironments 30. Single-cell RNA sequencing has enabled high-resolution identification of disease-associated cell populations and transcriptional states, but tissue dissociation removes information about their anatomical position, cellular neighbourhoods and structural context. Single-cell atlases of severe viral lung injury have further identified profibrotic macrophage and epithelial states associated with aberrant repair and fibrotic remodelling 146, 147. Spatial transcriptomic and related spatial profiling approaches complement these data by localizing disease-associated states within fibroblastic foci, aberrantly remodelled airway regions, honeycomb cysts, lymphoid aggregates, vascular niches and relatively preserved alveolar parenchyma 31, 37, 96, 145. The integration of single-cell and spatial information therefore links cellular heterogeneity to local pathological architecture and provides a basis for identifying multicellular niches rather than isolated disease-associated populations.

Fibroblast heterogeneity illustrates this principle particularly well 148-150. In the healthy lung, distinct fibroblast populations occupy specialized anatomical compartments, including the alveolar interstitium, peribronchial regions, perivascular adventitia, perichondrial regions and immune-cell-enriched stromal domains, where they contribute to ECM homeostasis, trophic support and immune regulation 16. During fibrosis, these populations do not simply converge towards a uniform 'activated fibroblast' phenotype. Instead, injury generates multiple disease-associated fibroblast states with differing inflammatory, immune-regulatory and matrix-remodelling programmes, including inflammatory and immune-recruiting fibroblasts as well as CTHRC1^+^ matrix-producing fibroblasts and myofibroblast states 37, 96, 151. Their abundance and localization vary across anatomical regions and disease contexts, indicating that fibroblast activation is better understood as a niche-dependent and dynamic state transition than as a fixed terminal phenotype. Local epithelial dysfunction, immune-cell composition, ECM remodelling and mechanical cues are likely to cooperate in shaping these spatially restricted fibroblast programmes 36, 37, 96.

This spatial heterogeneity also creates an important translational challenge. Single-cell studies have identified broadly comparable fibroblast programmes in human and murine lungs, with several orthologous markers shared across species, including *NPNT/Npnt*, *PI16/Pi16*, *CTHRC1/Cthrc1* and *CCL19/Ccl19* 10, 33. However, transcriptional similarity does not necessarily imply equivalent abundance, anatomical localization, lineage origin or behaviour during injury. Indeed, the trajectories through which these populations emerge and persist can differ substantially between chronic human pulmonary fibrosis and acute or partially resolving murine injury models. To clarify these similarities and limitations, Table 2 summarizes the major fibroblast populations identified in human and mouse lungs, together with representative molecular markers and proposed biological functions. This cross-species comparison distinguishes conserved stromal programmes from species- and model-dependent features that should be considered when extrapolating mechanistic findings from experimental systems to human pulmonary fibrosis.

#### Spatial omics technologies define the resolution of fibrotic microenvironmental mapping

Biological interpretation of fibrotic microenvironments is critically dependent on the spatial resolution, molecular coverage and preservation of tissue context afforded by the analytical platform. Tissue-dissociated single-cell RNA sequencing enables high-resolution characterization of cellular states and transcriptional programmes, but necessarily removes native spatial coordinates, cellular neighbourhoods and tissue architecture. Spatial omics approaches restore this anatomical dimension, although individual platforms differ substantially in their capacity to resolve single cells, delineate cellular boundaries, capture molecular diversity, survey tissue area and quantify spatial proximity 152-154. These differences directly constrain the biological inferences that can be drawn from spatial data. Current approaches used to interrogate pulmonary fibrosis can be broadly grouped into sequencing-based spatial transcriptomics, imaging-based spatial transcriptomics and region-of-interest (ROI)-based spatial profiling.

Sequencing-based spatial transcriptomic approaches, exemplified by 10x Genomics Visium, provide broad transcriptomic coverage and enable unbiased identification of disease-associated molecular domains. When integrated with single-cell reference atlases, these data can be computationally deconvolved to infer the spatial distribution of constituent cell populations using approaches such as cell2location 31, 145, 155. Conventional Visium capture spots, however, typically contain transcripts from multiple cells. Consequently, detection of macrophage, fibroblast and aberrant epithelial signatures within the same spot should primarily be interpreted as regional co-enrichment rather than evidence of direct cellular juxtaposition or functional communication. Higher-resolution sequencing-based approaches, including Visium HD, Slide-seq and Slide-seqV2, substantially reduce this spatial ambiguity and enable more refined delineation of fibrotic lesion boundaries, alveolar-fibrotic interfaces and narrow immune-stromal gradients 156-158. Nevertheless, their application to human IPF remains uneven across platforms. Whereas Slide-seq-based studies in human IPF remain relatively limited, Visium HD has recently been used to resolve molecular niches associated with aberrant epithelial structures, activated fibroblasts and distal lung remodelling in fibrotic tissue 145.

Imaging-based spatial transcriptomic platforms, including Xenium, MERFISH and CosMx, provide direct visualization of RNA molecules at single-cell or subcellular resolution. Their principal strength lies in resolving cellular boundaries and neighbourhood architecture, thereby enabling quantitative analysis of intercellular distances, cell-type adjacency and local multicellular organization 145, 159-161. For example, Xenium has been applied to spatially profile approximately 1.6 million cells across multiple human lung specimens, allowing reconstruction of epithelial, immune and fibroblast neighbourhoods within fibrotic tissue 145. By contrast, applications of MERFISH and CosMx to human IPF remain comparatively limited. These technologies therefore currently provide high-resolution platforms for validating candidate spatial relationships and defining local tissue architecture, although demonstration of functional communication still requires orthogonal perturbational or mechanistic evidence.

ROI-based platforms, such as GeoMx digital spatial profiling, provide a complementary strategy by enabling molecular comparison of histologically defined regions, including relatively preserved parenchyma, transitional zones and densely fibrotic lesions. Their compatibility with formalin-fixed paraffin-embedded specimens makes them particularly valuable for retrospective analysis of clinically annotated tissue collections 162. However, because molecular signals are aggregated across predefined regions, ROI-based approaches cannot independently establish whether specific cell populations are directly adjacent, organized within the same cellular neighbourhood or engaged in local cell-cell communication.

Collectively, the biological interpretation of spatial datasets must remain proportional to the resolution and measurement principles of the underlying platform. Region-level and capture-spot-based approaches are well suited to identifying the enrichment of cellular states and molecular programmes within pathological territories, whereas single-cell and subcellular-resolution methods provide stronger evidence for cellular adjacency, neighbourhood organization and local tissue architecture. Crucially, even high-resolution spatial proximity does not establish functional interaction. Spatial co-localization should therefore be viewed as evidence that constrains and prioritizes candidate cell-cell relationships, which must subsequently be validated using ligand-receptor analysis, multiplex imaging, *ex vivo* perturbation, lineage tracing or other mechanistic approaches.

#### Spatial immune-fibroblast interactions define the fibrotic niche

Although spatial omics has transformed the characterization of tissue microenvironments, spatial localization alone does not establish functional cell-cell communication or, by itself, define a pathological niche. Co-localization indicates spatial association but not direct interaction, whereas ligand-receptor co-expression identifies potential signalling relationships without demonstrating that signalling occurs in situ. Robust definition of immune-fibroblast interactions therefore requires an integrated evidence framework that combines candidate ligand-receptor inference, downstream transcriptional responses, spatial neighbourhood analysis and functional validation. Conceptually, this framework progresses from molecular compatibility, through spatial opportunity, to functional causality.

CellChat and CellPhoneDB provide a first layer of this framework by identifying candidate communication networks from single-cell transcriptomic data. Both infer potential interactions from ligand expression in putative sender populations and cognate receptor expression in recipient populations, but differ in their analytical architecture. CellChat aggregates ligand-receptor interactions into signalling pathways and estimates communication probabilities and network-level sender and receiver roles 163, 164. CellPhoneDB instead uses permutation-based testing to identify statistically enriched ligand-receptor pairs and explicitly accounts for multimeric ligand and receptor complexes 165. In pulmonary fibrosis, these approaches have revealed extensive candidate communication between macrophages and stromal populations. For example, CellChat analysis of human IPF lungs identified enhanced predicted communication between disease-associated macrophages and fibroblasts, myofibroblasts and epithelial cells across pathways including MIF, annexin, galectin, visfatin, midkine and CXCL signalling 166. Such observations nominate candidate profibrotic communication networks but remain transcriptomic predictions rather than evidence that the corresponding cell populations are juxtaposed or signalling to one another within intact tissue.

The value of these computational predictions increases when they are followed by functional perturbation. In another study, CellChat identified fibroblasts and myofibroblasts as major sources of CXCL12 and monocyte-derived alveolar macrophages as CXCR4-expressing recipients. Subsequent experiments showed that CXCL12 promoted macrophage recruitment and M2-like polarization, whereas pharmacological CXCR4 inhibition with AMD3100 or CXCR4 knockdown attenuated these responses; activated macrophages in turn enhanced fibroblast activation 167. This study therefore progressed from computational prediction to functional support for a reciprocal profibrotic circuit. Nevertheless, high-resolution spatial validation is still required to establish whether CXCL12-producing fibroblasts and CXCR4-expressing macrophages reproducibly occupy the same cellular neighbourhoods in human IPF. CellPhoneDB has similarly identified candidate interactions among endothelial, myeloid and stromal populations in experimental pulmonary fibrosis. In bleomycin-induced fibrosis, *Cxcl12*-expressing endothelial cells were predicted to engage CXCL12-CXCR4, FGF9-FGFR1 and collagen-integrin axes associated with monocyte recruitment, fibroblast proliferation and ECM remodelling 168. Again, such predictions define mechanistically plausible communication routes but do not independently establish their spatial execution *in situ*.

Ligand-target inference provides a second layer of evidence by asking whether candidate extracellular signals can account for transcriptional reprogramming in recipient populations. NicheNet extends conventional ligand-receptor analysis by integrating prior knowledge of ligand-receptor interactions, intracellular signalling and transcriptional regulatory networks to link sender-derived ligands with observed gene-expression programmes in recipient cells 169. In pulmonary fibrosis, this framework can be used to test whether immune-cell-derived ligands explain fibroblast programmes associated with matrix synthesis, migration and myofibroblast differentiation, or conversely whether fibroblast-derived signals account for immune-cell recruitment, survival or state transitions. In one IPF study examining the context of *MUC5B* risk variants, NicheNet analysis identified candidate signals from alveolar macrophages, fibroblasts and other microenvironmental populations that could contribute to transcriptional changes in alveolar type 1 epithelial cells, including IL-6 and amphiregulin, with selected ADAM17-associated patterns subsequently examined by immunofluorescence 170. Although this analysis did not specifically address immune-fibroblast coupling, it illustrates how ligand-target modelling can connect candidate sender populations to downstream responses in defined recipient cells.

More direct support for immune-fibroblast communication comes from studies integrating ligand-target inference with temporal, spatial and functional evidence. During the emergence of *Sfrp1^+^* intermediate fibroblasts and *Cthrc1^+^* myofibroblast states, NicheNet identified myeloid-derived TGF-β1 as a candidate upstream regulator of the *Cthrc1^+^* transcriptional programme. When combined with time-resolved single-cell trajectories, spatial mapping, chemokine-expression patterns and functional assays showing TGF-β1-induced fibroblast differentiation and invasive behaviour, these findings supported a reciprocal model in which fibroblasts recruit myeloid cells through chemokine signals and myeloid-derived TGF-β1 promotes progression towards an invasive Cthrc1^+^ myofibroblast state 150. This type of multimodal integration moves beyond simple ligand-receptor co-expression towards mechanistically directed hypotheses. However, NicheNet predictions remain dependent on prior regulatory networks and transcriptomic measurements and therefore still require spatial and experimental validation.

Spatial neighbourhood analysis provides the third layer by determining whether predicted sender and recipient populations have the physical opportunity to interact within native tissue. Spatial coordinates locate cells or molecular signals within tissue architecture, whereas the associated molecular information maps ligands, receptors and downstream programmes onto those positions. Integrating these dimensions makes it possible to test whether candidate signalling relationships are preferentially embedded within specific cellular neighbourhoods rather than arising from tissue-wide co-expression.

Tools such as Squidpy and Giotto reconstruct spatial neighbourhoods from cellular or capture-point coordinates and use neighbourhood enrichment, spatial autocorrelation and permutation-based statistics to determine whether particular cellular combinations occur more frequently than expected by chance 171, 172. Such statistical control is especially important in pulmonary fibrosis because disease-associated populations often undergo marked expansion 157, 173; apparent co-localization can therefore arise simply from increased abundance rather than selective spatial organization 174. Moreover, there is no universal definition of a spatial neighbourhood. Depending on platform resolution, tissue architecture and the biological scale under investigation, neighbourhoods may be defined by direct contact, fixed-distance radii, Delaunay graphs or k-nearest-neighbour relationships 171, 172, 175. Appropriate null models — for example, cell-label permutation or tissue-constrained spatial randomization — are therefore essential for determining whether observed adjacency, intercellular distances or local enrichment exceed expectations generated by cell abundance and anatomical compartmentalization. A biologically meaningful neighbourhood should consequently be reproducible across patients, tissue sections or anatomically comparable regions and robust to reasonable variation in analytical parameters.

Once candidate signalling relationships and statistically enriched cellular neighbourhoods have been established, fibrotic niches can be delineated using three complementary spatial strategies: pathology-guided regional classification, local cellular neighbourhood analysis and data-driven identification of spatial domains. Pathology-guided approaches use recognizable tissue structures — such as fibroblastic foci, aberrantly remodelled airway regions, honeycomb cysts or fibrotic-alveolar interfaces — as spatial reference units. Using fibroblastic foci as anchors, Franzén and colleagues compared lesion cores, peripheral regions and adjacent alveolar areas and identified spatial variation in fibroblast states, macrophage accumulation and TGF-β-, YAP/TEAD- and ECM-associated programmes across fibrotic interfaces 31. This strategy preserves direct histopathological interpretability but makes niche boundaries dependent on predefined morphological annotation.

Cell-centred neighbourhood approaches instead quantify the populations and molecular programmes surrounding individual anchor cells. Vannan and colleagues used spatial single-cell data to construct local niches from the 25 nearest neighbouring cells and integrated a 60-μm neighbourhood analysis with GraphSAGE-based modelling to identify molecular neighbourhoods 145. Such parameters should not be interpreted as universal biological boundaries. Rather, different spatial scales may capture distinct processes, ranging from direct cell contact to short-range paracrine signalling and broader tissue organization. Neighbourhood definitions should therefore be matched to platform resolution and biological question, with sensitivity analyses used to test the robustness of inferred spatial relationships.

Data-driven approaches identify spatial domains without imposing predefined anatomical boundaries. Methods including non-negative matrix factorization, spatial clustering and graph-based machine learning can detect recurrent combinations of cell states and molecular programmes directly from spatial datasets. Mayr and colleagues integrated cell2location-based deconvolution of Visium data with non-negative matrix factorization to identify fibrotic, airway macrophage and lymphoid immune niches, followed by validation using multiplex RNA in situ hybridization and protein imaging 30. Squidpy-based neighbourhood enrichment further revealed extensive disruption of normal alveolar organization in IPF, with fibrotic niches displaying spatial adjacency to alveolar, airway, immune and fibroblast-associated domains 30. These observations indicate that pathological niches are not randomly distributed but are embedded within interconnected spatial networks that can organize local immune-stromal communication.

Collectively, these analytical levels define an evidence hierarchy for assigning biological meaning to spatial immune-fibroblast interactions. Ligand-receptor inference establishes molecular compatibility; ligand-target analysis links candidate signals to recipient-cell responses; neighbourhood analysis establishes spatial opportunity; and perturbational or orthogonal validation provides evidence for functional causality. Pathology-guided, cell-centred and data-driven approaches then determine how these interactions are organized into reproducible tissue domains. A fibrotic niche should therefore not be defined solely by the co-occurrence of disease-associated cell types, but by the reproducible convergence of characteristic cellular composition, spatial organization, molecular signalling programmes and functionally supported intercellular interactions. This integrated definition provides a more rigorous basis for distinguishing true pathological niches from passive regional co-enrichment and for determining how immune-fibroblast circuits become spatially stabilized during pulmonary fibrosis.

#### Spatial composition and dynamic maintenance of fibrotic niches

Spatial omics studies have demonstrated that disease-associated cell states in pulmonary fibrosis are not randomly distributed, but instead assemble into recurrent local neighbourhoods with characteristic cellular compositions and molecular programmes. Across independent studies, neighbourhood-enrichment and permutation-based analyses have shown that pathological fibroblasts, aberrant epithelial states and profibrotic immune populations occur in spatial configurations more frequently than expected under appropriate null models 30, 31, 145. When integrated with ligand-receptor inference, downstream transcriptional responses and functional perturbation, these observations support the coordinated participation of these populations in fibrotic niche formation. However, regional co-enrichment, statistically significant spatial adjacency and functional cell-cell communication represent distinct levels of evidence and should not be considered interchangeable.

CTHRC1^+^ myofibroblasts constitute a prominent stromal component of these niches. In human fibrotic lungs, they are enriched within fibroblastic foci and regions of active tissue remodelling and represent one of the most reproducible pathological fibroblast states identified by single-cell and spatial studies 30, 31, 36, 145. Aberrant epithelial populations show a similarly non-random distribution. KRT8^+^ alveolar differentiation intermediates and KRT5^-^/KRT17^+^ basaloid-like epithelial cells accumulate within regions of disrupted alveolar architecture and frequently exhibit regional co-enrichment or statistically enriched proximity to pathological fibroblast states 30, 129, 176. These observations support a model in which failed alveolar regeneration is spatially coupled to persistent stromal remodelling. Whether such epithelial-fibroblast adjacency reflects sustained functional communication, however, requires additional evidence linking spatial proximity to directional signalling, downstream pathway activation and perturbation-sensitive phenotypes.

Immune populations form an additional organizational layer within the fibrotic niche. Among the best-supported spatial programmes is the association between SPP1^hi^ macrophages and pathological fibroblast populations enriched for *CTHRC1* and matrix-associated genes such as *POSTN* 32, 36, 177, 178. At single-cell spatial resolution, these populations can occur in closer proximity than expected by chance, suggesting preferential organization within the same pathological neighbourhoods. Candidate communication pathways include SPP1-CD44 and SPP1-integrin interactions, and experimental studies indicate that osteopontin can promote fibroblast migration, proliferation and matrix production 32, 179. Taken together, these findings support a model in which SPP1^hi^ macrophages contribute to the persistence of matrix-producing fibroblast states. Nevertheless, spatial adjacency and ligand-receptor compatibility remain insufficient to establish direct causal signalling in human IPF, which will require in situ interaction mapping together with genetic, pharmacological or *ex vivo* perturbation.

Fibrotic niche maintenance depends not only on cellular composition, but also on persistent local signalling and progressive remodelling of the extracellular environment. Aberrant epithelial cells, pathological fibroblasts and profibrotic immune populations participate in signalling networks involving TGF-β, IL-1β, TNF, Notch and other context-dependent pathways that can reinforce fibroblast activation, sustain abnormal epithelial states and amplify local inflammatory responses 34, 35. In parallel, continued ECM deposition, collagen crosslinking and increasing tissue stiffness enhance integrin-dependent mechanotransduction and facilitate activation of matrix-sequestered latent TGF-β. These mechanical and biochemical inputs further stabilize pathological stromal states, creating reciprocal feedback between cellular communication and the ECM. The niche is therefore maintained not simply by persistent cells, but by continual coupling between cellular state, local signalling and matrix mechanics.

Consistent with this model, IPF lungs contain multiple spatially distinct but interconnected pathological microenvironments rather than a single uniform fibrotic compartment. These include SPP1^+^ macrophage-enriched regions, lymphoid aggregates, remodelled vascular compartments and stromal populations associated with tertiary lymphoid structures 145, 180. Such niches are likely to differ in their dominant cellular constituents, signalling programmes and degree of matrix remodelling, while remaining functionally connected across the diseased tissue. Pulmonary fibrosis can therefore be viewed as a network of interacting microenvironments in which local epithelial, immune and stromal abnormalities become progressively coordinated rather than as a spatially homogeneous process (Figure 4).

A major limitation of the current evidence is its predominantly cross-sectional nature. Most spatial studies of human IPF analyse explanted or otherwise advanced-stage tissue and therefore capture established pathological architecture more effectively than the processes through which individual niches emerge, expand and mature 30, 31, 145. Cross-sectional spatial gradients and inferred cellular trajectories can suggest possible sequences of niche evolution, but they cannot independently establish temporal ordering. Progress will require longitudinal or stage-resolved human sampling, integration of spatial transcriptomics with spatial proteomics and multiplex imaging, and experimental systems in which cell-state transitions can be followed while tissue architecture is preserved. RNA velocity and trajectory inference may provide hypotheses regarding state transitions, whereas lineage tracing and targeted perturbation are required to establish lineage relationships and causal mechanisms. Combining these approaches should enable reconstruction of niche initiation, maintenance and expansion across disease progression.

From this perspective, spatiotemporal locking is not defined by simple persistence or co-localization of pathological cell populations. Rather, it emerges through the convergence of three features: first, reproducible spatial organization of disease-associated epithelial, immune and fibroblast states; second, sustained functional coupling through directional signalling and corresponding recipient-cell responses; and third, integration of these cellular circuits with ECM remodelling, collagen crosslinking and mechanotransduction that stabilizes pathological programmes beyond the period of the initiating injury. Temporal persistence creates the opportunity for pathological states to endure, whereas spatial organization and matrix feedback constrain their capacity to revert. When these processes become mutually reinforcing, the fibrotic niche acquires a stable local architecture that resists restoration of tissue homeostasis. This convergence provides a mechanistic definition of spatiotemporal locking and links the dynamic maintenance of fibrotic niches to the progressive loss of tissue plasticity.

## Molecular frameworks of reciprocal immune-fibroblast communication

### Soluble mediators and signalling hierarchies

The persistence of fibrotic niches is not sustained by a single signalling pathway, but by interconnected molecular layers that couple immune activation to stromal reprogramming. Soluble mediators, ECM remodelling and mechanotransduction, together with metabolic and epigenetic programmes, reinforce pathological cell states and progressively reduce their capacity to return towards homeostasis. These regulatory layers interact through feed-forward and feedback circuits that stabilize local cellular organization and contribute to spatiotemporal locking of the fibrotic niche. Among them, soluble mediators provide the most immediate interface for reciprocal immune-fibroblast communication, linking tissue injury and inflammatory amplification to fibroblast activation, persistence and functional specialization.

TGF-β occupies a central position within this signalling network. It can be produced by multiple cellular compartments, including macrophages, lymphocytes, injured epithelial cells and activated fibroblasts, but its biological activity depends on considerably more than transcript or protein abundance. Cellular source, timing, anatomical localization, mechanisms of latent TGF-β activation and the signalling state of the responding cell collectively determine its effects. Epithelial-derived TGF-β can promote activation and myofibroblast differentiation of neighbouring fibroblasts 31, 135, 181, whereas macrophage-derived or locally activated TGF-β can reinforce pathological fibroblast states, ECM deposition and fibrotic expansion within immune-stromal niches 29, 182, 183. Single-cell and spatial studies further reveal heterogeneous TGF-β-response programmes among fibroblast populations 31, 36, 37. However, direct evidence that distinct cellular sources of TGF-β selectively instruct specific fibroblast subsets remains limited. Current data therefore favour a context-dependent model in which TGF-β responses emerge from the intersection of ligand source, local activation, disease stage, spatial context and recipient-cell state rather than from fixed source-to-subset relationships. Resolving these relationships will require source-specific genetic perturbation combined with spatially resolved analyses.

Once activated, TGF-β engages canonical SMAD signalling together with non-canonical pathways, including MAPK, PI3K-AKT and Rho-family GTPase signalling, thereby promoting fibroblast proliferation, survival, contractility, collagen synthesis and myofibroblast persistence 184, 185. Importantly, TGF-β signalling is embedded within a broader cytokine network. IL-4 and IL-13 couple type 2 immunity to stromal activation through receptor complexes containing IL-4Rα and activation of JAK-STAT signalling, particularly STAT6, promoting fibroblast proliferation, matrix synthesis and myofibroblast differentiation 52. TNF and IL-1β can further modify fibroblast behaviour by amplifying inflammatory signalling, altering chemotaxis and matrix turnover, and cooperating with profibrotic pathways in a context-dependent manner 34. In parallel, PDGF, FGF ligands, EGF receptor ligands and amphiregulin provide proliferative and survival signals that support expansion and maintenance of activated stromal populations 186-189. Fibroblast fate therefore reflects integration of multiple simultaneous inputs rather than the action of an isolated cytokine.

The relative importance of these signals changes across disease progression and anatomical context 190. During early injury, inflammatory cytokines, chemokines and monocyte-recruiting signals are prominent components of the reparative response. If resolution fails, persistent immune-derived signals increasingly converge with TGF-β activity, fibroblast-autonomous programmes and ECM-derived mechanical cues. In established fibrosis, matrix stiffness, latent TGF-β activation and immune-suppressive stromal circuits can become increasingly important for maintaining pathological fibroblast states 191. Thus, signalling hierarchies are not fixed: the same mediator can have different consequences depending on when and where it is produced, how it is activated and which cellular state receives the signal.

This context dependence has direct therapeutic implications. The failure of broad immunosuppressive strategies in IPF should not be interpreted as evidence that immune mechanisms are irrelevant to fibrosis. Rather, it highlights the limitations of suppressing immune activity without accounting for disease stage, cellular source, spatial niche and the transition from inflammation-dependent repair to stromal- and matrix-reinforced disease 192, 193. A more informative therapeutic framework is therefore to identify which signalling circuits are dominant within particular pathological niches and at specific stages of disease, and to determine when disruption of an immune-fibroblast interaction can destabilize fibrosis without compromising protective immunity or physiological tissue repair.

### Matrix mechanics, mechanotransduction and mechanical memory

The ECM is not merely an end product of fibroblast activity, but an active signalling platform that stores biochemical ligands, transduces mechanical forces and shapes the behaviour of both stromal and immune cells. In pulmonary fibrosis, progressive matrix accumulation, crosslinking and stiffening transform the lung from a mechanically permissive repair environment into a tissue state that actively reinforces fibrosis. Increased matrix stiffness enhances fibroblast activation, supports myofibroblast persistence and can modify the activation and migratory behaviour of immune cells 194, 195. Thus, matrix mechanics constitute an additional regulatory layer through which initially cellular and soluble signalling programmes become structurally embedded within the fibrotic niche.

Persistent increases in ECM stiffness reflect not simply excessive collagen deposition, but a broader imbalance between matrix degradation, deposition and crosslinking. Under physiological repair conditions, matrix metalloproteinases (MMPs) and tissue inhibitors of metalloproteinases (TIMPs) coordinate matrix turnover and tissue remodelling. In pulmonary fibrosis, however, this system is not uniformly activated or suppressed; rather, individual MMPs and TIMPs exhibit distinct cellular sources, substrates and temporal functions, and their collective dysregulation can favour ECM retention and aberrant restructuring 196-198. In human IPF lungs, for example, TIMP-1 has been localized predominantly to interstitial macrophages, TIMP-2 to fibroblastic foci and TIMP-3 to vascular elastic structures, illustrating the spatial compartmentalization of matrix-regulatory programmes. Bleomycin-induced fibrosis is similarly associated with early and sustained increases in TIMP-1, consistent with the possibility that excessive local protease inhibition limits effective matrix clearance 196, 199.

The functions of MMPs are equally context dependent. Although MMP-2 and MMP-9 are frequently increased in fibrotic lungs, their elevation does not necessarily translate into effective collagen clearance. MMP-9 can contribute to basement-membrane remodelling, fibroblast migration and activation of latent TGF-β, thereby potentially reinforcing rather than resolving fibrosis 200. By contrast, MMP-13 participates directly in fibrillar collagen degradation, and loss of *Mmp13* exacerbates bleomycin-induced fibrosis, supporting a protective role for selected collagenolytic pathways 201. Consistent with an imbalance between proteolysis and inhibition, a study of human interstitial lung disease reported a decline in the bronchoalveolar-lavage-fluid MMP-9/TIMP-1 ratio with increasing fibrosis severity and an association with fibroblastic foci 202. Activated fibroblasts and myofibroblasts may contribute to this altered protease-inhibitor balance, further shifting local matrix turnover towards persistence rather than restoration.

Matrix stabilization is reinforced by covalent crosslinking. Lysyl oxidase (LOX) and lysyl oxidase-like (LOXL) family members catalyse crosslinking of collagen and elastin, converting relatively compliant matrices into mechanically stabilized structures 203, 204. Multiple family members, including LOX, LOXL2, LOXL3 and LOXL4, have been implicated in human IPF and experimental pulmonary fibrosis, with expression of selected isoforms enriched in activated fibroblasts and fibroblastic foci 205. Pharmacological and genetic studies further suggest that these enzymes are not functionally equivalent. Inhibition of LOXL2 or LOXL3 can reduce abnormal collagen organization and tissue stiffening in experimental settings, whereas recent genetic studies indicate a prominent contribution of LOXL4 to pathological collagen crosslinking, with more limited effects reported following LOXL2 deficiency 206, 207. These observations argue that matrix stiffening is governed by partially overlapping but non-redundant enzymatic programmes rather than by a single crosslinking pathway.

Once established, a stiffened ECM actively feeds back onto fibroblast state. Persistent collagen crosslinking increases cell-matrix coupling and promotes integrin-dependent mechanotransduction, including mechanical activation of matrix-sequestered latent TGF-β 44, 207-209. Fibroblasts sense matrix properties through integrins, cadherins, CD44, discoidin domain receptors and mechanically activated ion channels such as TRPV4 and PIEZO1. These inputs converge on FAK, Rho-ROCK, MRTF-SRF and YAP-TAZ signalling, which regulate cytoskeletal tension, contractility and profibrotic transcriptional programmes 83, 210, 211. In this way, mechanical stress and canonical profibrotic signalling become reciprocally coupled: fibroblasts stiffen the matrix, and the stiffened matrix further stabilizes activated fibroblast states through mechanotransduction and TGF-β activation 212, 213.

A particularly important consequence of prolonged mechanical exposure is mechanical memory 214. Fibroblasts maintained on stiff matrices can acquire durable transcriptional, epigenetic and cytoskeletal adaptations that persist after the original mechanical stimulus is reduced or removed 215, 216. Such memory provides a potential explanation for the persistence of activated fibroblast programmes after the initiating inflammatory or mechanical insult has subsided. It also adds a temporal dimension to matrix signalling: fibroblast behaviour reflects not only the current mechanical environment, but also prior exposure to pathological stiffness. Mechanical memory may therefore contribute to the progressive loss of stromal plasticity and help consolidate the spatiotemporal locking of established fibrotic niches.

The consequences of matrix remodelling extend beyond fibroblasts to the immune compartment. Changes in ECM stiffness, architecture and composition can influence immune-cell migration and retention, alter chemokine presentation and modify activation thresholds in myeloid populations 217-220. As MMP-TIMP imbalance and LOX-family-mediated crosslinking drive progressive matrix restructuring, collagen fibres become denser and mechanically stabilized. Depending on matrix architecture and anatomical context, such changes can restrict cellular motility and access to densely remodelled regions while favouring retention of selected immune populations and soluble mediators 221-223. ECM components and glycosaminoglycans can additionally bind and concentrate mediators such as TGF-β and CXCL12, thereby regulating their local bioavailability and spatial gradients 224. The matrix therefore acts simultaneously as a physical barrier, a reservoir of biochemical signals and a regulator of their spatial distribution.

Through these combined biochemical and mechanical effects, the ECM can help organize aberrant epithelial cells, profibrotic macrophages and pathological fibroblasts into persistent local neighbourhoods 30, 31. By promoting the persistence and spatial organization of these pathological cell states, ECM remodelling can further stabilize fibrotic niches and may contribute to immune-privileged-like features in specific fibrotic regions. This does not imply that matrix architecture alone determines cellular organization, but rather that altered ECM provides a structural and signalling context in which reciprocal cellular interactions can be repeatedly reinforced. Conversely, immune cells actively reshape this context through MMPs, TIMPs and matricellular mediators, thereby modifying matrix composition and feeding back onto fibroblast behaviour 225, 226. The ECM should therefore be viewed as a bidirectional regulatory interface that couples immune activation, fibroblast state, tissue mechanics and spatial organization. By embedding prior cellular activity into a persistent structural environment, matrix remodelling provides a mechanism through which transient inflammatory and stromal responses can become mechanically stabilized within chronic fibrotic niches.

### Metabolic coupling and chronic inflammatory training

Immune-fibroblast crosstalk is also shaped by metabolic state. During pulmonary fibrosis, both activated fibroblasts and immune cells undergo substantial metabolic reprogramming, including changes in glycolysis, mitochondrial function, fatty-acid metabolism, amino-acid utilization and redox homeostasis 227-229. These adaptations are not merely secondary responses to cellular stress; they can influence cell fate, biosynthetic capacity and the composition of the local signalling milieu, thereby adding a metabolic layer to reciprocal immune-stromal communication 230, 231.

Macrophage and fibroblast states are particularly sensitive to these metabolic constraints. Disease-associated macrophages adopt metabolic programmes that differ according to activation state, tissue localization and stage of fibrosis rather than conforming to a single profibrotic metabolic phenotype 232. In parallel, activated fibroblasts increase the biosynthetic and energetic capacity required for proliferation, contractility and sustained ECM production, with glycolytic, mitochondrial and amino-acid pathways contributing to these demands 233. Because immune and stromal populations occupy the same spatially restricted niches, the metabolites generated or consumed by one population can alter the functional state of neighbouring cells. Lactate, lipid-derived mediators and changes in amino-acid availability may therefore function not only as metabolic by-products but also as local signalling cues that reinforce pathological cell states 39, 234, 235.

Lactate illustrates how metabolic remodelling can become integrated into profibrotic signalling. Increased glycolytic flux in activated stromal and inflammatory populations can elevate extracellular lactate, alter local pH and influence cellular transcriptional and epigenetic programmes. In fibroblasts, lactate-rich environments can favour matrix-producing phenotypes, whereas in immune cells lactate can modify inflammatory activation, polarization and effector function in a context-dependent manner. Lipid metabolism provides a parallel mechanism. Fibrotic tissues contain altered lipid species and lipid-handling programmes that can shape macrophage survival and state transitions, including TREM2-associated macrophage programmes, while bioactive lipid mediators can influence fibroblast migration, proliferation and matrix remodelling 39, 234, 235 . These observations suggest that metabolic communication within fibrotic niches is likely to involve both exchange of metabolites and reciprocal competition for limiting nutrients.

Metabolic state can also influence the persistence of pathological programmes through its interaction with chromatin regulation. Metabolites such as acetyl-CoA, α-ketoglutarate, succinate and NAD^+^ regulate enzymes involved in histone acetylation, DNA and histone demethylation and cellular stress responses, thereby linking nutrient availability to transcriptional stability. Persistent metabolic perturbation may therefore reinforce disease-associated macrophage and fibroblast states by altering the epigenetic landscape that governs their responsiveness to subsequent stimuli. In this sense, metabolism provides a potential bridge between transient environmental exposure and longer-lived cellular memory.

This concept intersects with trained immunity, in which prior stimulation induces durable metabolic and epigenetic reprogramming that alters the magnitude or quality of subsequent innate immune responses 236-238. Trained immunity is well established in myeloid cells, but whether an equivalent programme operates in lung fibroblasts requires greater caution. Fibroblasts can retain prolonged transcriptional and epigenetic consequences of inflammatory or mechanical exposure and may exhibit enhanced responsiveness upon restimulation, but these phenomena are more appropriately described at present as inflammatory or stromal memory rather than classical trained immunity unless durable stimulus-independent recall responses are demonstrated. Repeated injury may therefore generate parallel forms of pathological memory in immune and stromal compartments, with metabolic rewiring, chromatin remodelling and mechanical history jointly reducing the capacity of these cells to return towards homeostasis.

Such memory mechanisms are particularly relevant to ageing and chronic disease. Pulmonary fibrosis is strongly age-associated, and ageing alters mitochondrial fitness, redox balance, nutrient sensing, cellular senescence and epigenetic regulation in both immune and stromal populations. These age-related changes may lower the threshold for persistent activation and increase the probability that otherwise reversible responses become stabilized after repeated injury. Metabolic, epigenetic and mechanical memory can therefore be viewed as complementary mechanisms through which the history of tissue injury becomes encoded within cellular states and contributes to the long-term maintenance of fibrotic niches (Figure 5).

Taken together, the molecular circuits that sustain immune-fibroblast crosstalk are neither pathway-autonomous nor temporally fixed. Soluble mediators, matrix-derived mechanical cues, metabolic state and epigenetic memory interact in a stage- and niche-dependent manner to determine the behaviour of participating cells. The same mediator can therefore exert different effects according to its cellular source, local availability, anatomical context and recipient-cell state. TGF-β provides a clear example: transient TGF-β activity contributes to physiological wound repair, whereas persistent activation within a stiffened, inflammatory and metabolically altered niche can stabilize pathological fibroblast programmes. Similar context dependence applies to inflammatory cytokines, chemokines, immune-checkpoint pathways and metabolic signals.

Accordingly, a pathway-centred taxonomy alone is insufficient to capture the biological organization of fibrogenic signalling. Table 3 therefore summarizes major immune-fibroblast regulatory programmes according to their principal cellular sources and targets, dominant biological effects, spatial and temporal context, strength of supporting evidence and potential therapeutic window. These programmes include canonical profibrotic signals such as TGF-β, PDGF and IL-13; inflammatory pathways involving IL-1β, TNF and IL-17A; niche-associated communication axes such as SPP1-CD44 and CXCL12-CXCR4; immune-evasion pathways including PD-1-PD-L1 and CD47-SIRPα; and mechanical, metabolic and epigenetic programmes that stabilize pathological cell states. This framework emphasizes that fibrogenic pathways do not possess fixed pathogenic roles, but acquire their biological significance through the cellular, spatial and temporal context in which they operate.

## Translational constraints and therapeutic implications

### What current models reveal

Experimental models remain indispensable for dissecting stromal-immune interactions in pulmonary fibrosis, but no single system reproduces the tempo, heterogeneity, chronicity and limited reversibility of human IPF. The single-dose bleomycin model remains the most widely used and has generated substantial mechanistic insight into acute epithelial injury, inflammatory-cell recruitment, provisional matrix deposition and the subsequent fibrogenic response. Its relatively well-defined temporal course makes it particularly useful for resolving early immune-stromal interactions and for identifying the contributions of monocyte-derived macrophages, injury-responsive fibroblast states and mechanosensitive stromal circuits 239. However, its prominent acute inflammatory phase and tendency towards partial spontaneous resolution distinguish it from the persistent and heterogeneous remodelling characteristic of human IPF.

Models based on repeated bleomycin exposure, silica or asbestos provide complementary information by extending the duration of injury and more closely modelling persistent or exposure-driven fibrogenesis. These systems are therefore useful for interrogating chronic inflammatory signalling, sustained immune-cell recruitment, progressive ECM remodelling and the maintenance of fibrotic lesions. Genetic models provide a different dimension of disease biology by reproducing selected susceptibility mechanisms, including epithelial dysfunction, telomere instability, defective progenitor-cell responses and impaired alveolar regeneration 126, 128, 177, 240, 241. Rather than representing interchangeable models of the same disease process, these systems capture distinct components and temporal phases of fibrogenesis.

Human-derived experimental platforms address some of the limitations inherent to animal models. Precision-cut lung slices (PCLS) preserve native tissue architecture, multicellular composition and pre-existing ECM, enabling investigation of short-term cellular communication and pharmacological responses within human lung tissue. Lung organoids permit controlled interrogation of epithelial-mesenchymal interactions and regenerative programmes, whereas lung-on-chip systems provide tunable environments in which cellular composition and defined signalling inputs can be experimentally manipulated 242, 243. These platforms offer important advantages for examining human-specific responses, although each also sacrifices aspects of the systemic immune, vascular or temporal complexity present *in vivo*.

The value of individual models therefore depends on the biological question and disease stage being investigated. Single-dose bleomycin is particularly informative for acute epithelial injury, inflammatory recruitment and the transition from early repair towards fibrogenesis. Repeated-injury, silica- and asbestos-based models are better suited to questions involving persistent damage, chronic immune activation and progressive matrix remodelling. Genetic models are most informative for defining mechanisms of disease susceptibility and failed regeneration, whereas PCLS, organoids and lung-on-chip systems provide complementary platforms for testing human cell-state interactions and therapeutic responses. Integration of these models with single-cell transcriptomics, spatial profiling and lineage-tracing approaches can further resolve cell-state transitions, lineage relationships and the spatial organization of emerging fibrotic niches.

Taken together, experimental models have provided causal evidence that monocyte recruitment, macrophage-state transitions, fibroblast reprogramming and matrix feedback are active components of fibrogenesis rather than merely correlates of diseased tissue. They have also established that fibroblast phenotypes are dynamic and arise along trajectories shaped by immune-derived, epithelial-derived and matrix-derived cues. However, the strength of mechanistic inference depends on the model in which it is obtained. Findings from acute injury models are most informative for early-stage inflammatory and reparative mechanisms, whereas conclusions about niche persistence, fibrosis maintenance or reversal require validation in chronic models and, where possible, human-derived systems.

This distinction is equally important for therapeutic studies. Acute injury models are well suited to testing interventions that target inflammatory amplification or early immune-stromal coupling, but they may overestimate the efficacy of treatments administered before stable fibrotic architecture has developed. By contrast, chronic injury models and human-derived platforms such as PCLS are more appropriate for evaluating interventions directed at established fibroblast states, matrix persistence and remodelling reversal. Future translational studies should therefore adopt stage-matched, multi-model strategies in which the experimental system is selected according to the mechanism and therapeutic window under investigation. Combining complementary models across temporal and spatial scales will be essential for determining which immune-stromal interactions initiate fibrosis, which maintain established niches and which remain therapeutically reversible.

### Why translation remains difficult

Despite substantial mechanistic progress, translation from experimental models to clinical benefit has remained inconsistent. A major source of this gap is the mismatch between the biological context captured by many experimental systems and that encountered in human IPF. Common animal models are dominated by acute epithelial injury and inflammatory recruitment, whereas IPF typically develops in older individuals as a chronic, spatially heterogeneous and progressively dysregulated disease. The partial spontaneous resolution observed after single-dose bleomycin further contrasts with the limited reversibility of established human fibrosis 244. Moreover, defining pathological features of usual interstitial pneumonia, including persistent fibroblastic foci, extensive architectural distortion and marked heterogeneity among neighbouring lesions, are only incompletely reproduced in murine models 245.

This mismatch extends beyond disease kinetics. Human pulmonary fibrosis develops within a biological history that includes ageing, cumulative environmental exposures, genetic susceptibility, metabolic stress, comorbidities and substantial interpatient molecular heterogeneity. These variables influence epithelial regenerative capacity, immune-cell state, fibroblast behaviour and ECM remodelling, yet are difficult to reproduce simultaneously in experimental systems. Consequently, an intervention that suppresses an early inflammatory programme in a young experimental animal may have limited efficacy when administered to a patient with an established, mechanically stabilized and immunologically remodelled fibrotic niche. Translational failure may therefore reflect not only insufficient target engagement, but also stage mismatch, biological mismatch and niche mismatch between experimental intervention and clinical disease.

A related problem is that therapeutic efficacy is often evaluated at different biological stages in experimental and clinical settings. Preventive or early therapeutic dosing in animal models frequently precedes the establishment of stable fibrotic architecture, whereas patients generally enter clinical trials after fibrosis is already detectable and, in many cases, structurally entrenched. Interventions directed against inflammatory recruitment may therefore appear highly effective in preclinical studies yet show limited benefit once fibrosis has become maintained by fibroblast persistence, ECM stiffening and self-reinforcing stromal-immune circuits. Similarly, reduction in collagen content or histological injury over short experimental intervals does not necessarily predict durable restoration of lung architecture or function in chronic human disease.

These limitations do not diminish the value of experimental models; rather, they define the conditions under which their findings should be interpreted. Mechanistic conclusions should be matched to the temporal stage, cellular context and pathological process represented by the model, and translational claims should distinguish evidence for disease initiation from evidence for maintenance or reversal of established fibrosis. A treatment that prevents fibrogenesis should not automatically be assumed to reverse an established fibrotic niche.

An important priority is therefore to develop and combine experimental systems that explicitly address the spatiotemporal dimensions of fibrosis. Repeated-injury and ageing models can better interrogate chronic state stabilization; longitudinal or stage-resolved sampling can distinguish initiation from maintenance; patient-derived organoids and precision-cut lung slices can test human-specific cellular responses; and organ-chip platforms can experimentally manipulate mechanical and multicellular variables. Integration of these systems with single-cell and spatial profiling of human biopsy, explant and bronchoalveolar lavage samples should allow experimental mechanisms to be aligned more precisely with corresponding human disease states. The goal is not to identify a single model that reproduces IPF in its entirety, but to construct a translational evidence chain in which complementary models interrogate the appropriate disease stage, niche architecture and therapeutic question.

### Targeting cellular interactions rather than isolated cell types

A major therapeutic implication of the emerging niche-based model is that durable antifibrotic activity may require disruption of pathological cellular interactions rather than depletion or inhibition of individual cell populations in isolation 145. Current antifibrotic therapies, including nintedanib and pirfenidone, attenuate multiple profibrotic processes and slow disease progression, but they do not specifically dismantle the multicellular circuits that stabilize established fibrotic niches 246, 247. Table 4 places approved therapies together with representative investigational and immune-directed approaches in their broader translational context. These interventions act across diverse biological processes, including VEGFR, FGFR and PDGFR signalling, TGF-β activation, PDE4B-dependent inflammatory and fibrotic pathways, LPA1 signalling, CTGF-associated matrix remodelling, macrophage-associated programmes and adaptive immune activation. Collectively, this therapeutic landscape illustrates both the progress achieved by broad antifibrotic strategies and the continuing need for interventions that selectively disrupt pathogenic immune-fibroblast circuits while preserving host defence and physiological repair 25, 31.

One therapeutic opportunity is to disrupt macrophage-fibroblast coupling 248. Candidate targets include SPP1-CD44 signalling, CSF1-dependent macrophage maintenance, MERTK-associated myeloid programmes and pathways governing monocyte recruitment 32, 136, 249. These axes are attractive because they occupy upstream positions within fibrogenic niches: macrophage persistence sustains fibroblast activation, while fibroblast-derived signals can reciprocally reinforce pathological macrophage states. However, their translational maturity varies substantially. SPP1-CD44, MERTK and monocyte-recruitment strategies remain supported predominantly by human tissue associations and preclinical mechanistic studies, whereas CSF1-CSF1R targeting has broader clinical development experience in oncology and selected fibrotic conditions 24, 32, 250, 251. Even for the more clinically tractable pathways, therapeutic design will need to distinguish pathogenic macrophage states from macrophage functions required for host defence, efferocytosis and tissue repair 252. Temporal and cellular selectivity are therefore likely to be as important as target selection itself.

A second opportunity is to restore immune surveillance of pathological stromal cells. Preclinical studies implicating PD-1-PD-L1, CTLA-4 and CD47-related pathways suggest that dysfunctional adaptive immunity and impaired clearance of activated fibroblasts may contribute to fibrosis persistence 63-65. These findings raise the possibility that selected immune-checkpoint pathways could be therapeutically manipulated to enhance stromal clearance. However, this concept remains substantially less mature in pulmonary fibrosis than in oncology. Immune checkpoints regulate fundamental aspects of immune tolerance and activation, and systemic blockade could provoke excessive inflammation or immune-mediated tissue injury. Translation will therefore require a much higher degree of precision than simple checkpoint inhibition, potentially through cell-restricted delivery, transient modulation or biomarker-guided intervention during disease stages in which failed immune surveillance is demonstrably contributing to fibrosis.

A third strategy is to target pathological fibroblast states while preserving reparative stromal functions. Single-cell and spatial studies have identified CTHRC1^+^ matrix-producing fibroblasts, inflammatory fibroblast populations and stromal cells associated with tertiary lymphoid structures as candidate disease-associated states 144. In principle, such populations offer greater specificity than targeting fibroblasts as a single compartment. However, the translational challenge is substantial: most proposed states are defined transcriptionally, their lineage relationships and stability remain incompletely resolved, and few currently have surface markers or dependencies that can be selectively exploited in patients. Thus, fibroblast-state-selective therapy remains primarily a conceptual and preclinical strategy. Progress will depend on identifying stable, accessible and functionally necessary features of pathological fibroblast states and developing delivery systems capable of exploiting them without disrupting fibroblasts that support alveolar homeostasis or regeneration 253.

A fourth therapeutic axis targets matrix mechanics and mechanotransduction. Integrins, Rho-ROCK pathways, YAP-TAZ signalling and collagen-crosslinking enzymes represent candidate intervention points within the structural feedback loops that sustain myofibroblast activation 254-256. In contrast to strategies aimed principally at inflammatory initiation, matrix-directed approaches may be particularly relevant once fibrosis has become mechanically stabilized. Integrin-directed and Rho-ROCK-modulating approaches have progressed into clinical evaluation in IPF or other fibrotic diseases, whereas direct targeting of YAP-TAZ programmes and selected collagen-crosslinking pathways remains predominantly preclinical 257-259. Because mechanotransduction is also required for normal tissue repair and regeneration, efficacy will depend on identifying therapeutic windows in which pathological mechanical reinforcement can be weakened without compromising physiological wound healing.

Metabolic and epigenetic programmes provide an additional layer of therapeutic opportunity. Pathological immune and fibroblast states depend on altered energetic, biosynthetic and chromatin-regulatory programmes, suggesting that metabolic intervention could reduce the persistence of disease-associated cell states rather than simply suppress a single extracellular signal 230. The major challenge is selectivity: many of the relevant metabolic pathways are fundamental to normal cellular function. Their therapeutic potential may therefore lie in combination strategies that lower the stability of pathological states and sensitize them to more targeted antifibrotic interventions rather than in complete systemic inhibition of core metabolic pathways.

These candidate strategies differ markedly in both evidential support and translational maturity (Table 5). Integrin-directed approaches have undergone relatively direct clinical evaluation in IPF and therefore have a more immediate translational evidence base. By contrast, SPP1-CD44, MERTK, PD-1-PD-L1, CD47 and YAP-TAZ targeting is supported mainly by mechanistic studies, experimental models and human tissue associations, and requires further validation of disease-stage specificity, targetable cell populations and long-term safety. CSF1-CSF1R, Rho-ROCK and metabolic interventions occupy an intermediate position: these pathways have broader clinical or pharmacological development experience across diseases, but their optimal therapeutic window and benefit-risk profile in IPF remain uncertain.

Therapeutic prioritization should therefore not be based solely on whether inhibition of a pathway reduces fibrosis in an experimental model. A more rigorous framework should consider at least five dimensions: causal evidence, disease-stage relevance, spatial niche context, translational maturity and consequences for physiological repair. Targets supported only by spatial association or transcriptomic inference require different development strategies from those validated by genetic perturbation and human pharmacology. Likewise, an intervention suitable for early inflammatory amplification may be poorly matched to established matrix-dominant fibrosis. A niche-based therapeutic framework therefore shifts the central question from which molecule is profibrotic? to which interaction is necessary for the maintenance of a particular pathological niche, at which disease stage, and can it be disrupted without destabilizing physiological tissue function?

### Combinatorial and stage-specific therapy

Because pulmonary fibrosis is sustained by interacting epithelial, immune, stromal and matrix abnormalities, combination therapy may ultimately prove more effective than interventions directed at a single pathway or cell population 260. A key principle is temporal matching of therapy to disease state. During early injury and failed repair, treatment may need to limit excessive inflammatory amplification while preserving host defence and regenerative responses; once fibrosis becomes established, therapeutic emphasis may need to shift towards disrupting pathological stromal persistence, matrix reinforcement and immune exclusion 226, 261, 262. Thus, effective combination therapy is unlikely to consist simply of adding multiple antifibrotic agents, but rather of targeting different components of the fibrotic network at the stages when they are most functionally relevant.

This principle is particularly important for macrophage-directed therapy. During acute injury and early repair, resident and recruited macrophages contribute to debris clearance, efferocytosis, inflammatory resolution and epithelial regeneration 263, 264. Non-selective inhibition of CSF1R, MERTK or monocyte recruitment during this phase could therefore interfere with reparative macrophage functions and delay restoration of tissue homeostasis. By contrast, persistent injury can stabilize disease-associated macrophage programmes, including SPP1^+^ states, and reinforce macrophage-fibroblast coupling within established fibrotic niches. At this stage, selective disruption or reprogramming of pathological macrophage states may provide a more favourable therapeutic window. The objective should therefore be not indiscriminate macrophage depletion, but state- and stage-selective modulation of macrophage programmes that are necessary for maintenance of the fibrotic niche.

A complementary strategy is biomarker-guided precision therapy, in which patients are stratified according to the dominant biological programmes operating within their disease. Candidate niche patterns might include SPP1-associated macrophage-fibroblast coupling, tertiary-lymphoid-structure-rich adaptive immune remodelling, fibroblast-dominant matrix production or mechanically stabilized fibrosis 30, 265, 266. Such stratification will require biomarkers that capture different levels of disease biology rather than relying on a single circulating analyte or imaging feature.

Circulating, tissue-derived and imaging biomarkers may provide complementary information about these biological states. Increased circulating SPP1 may reflect enhanced activity of SPP1-associated macrophage programmes, whereas CTHRC1, POSTN and other matrix-associated markers may indicate pathological fibroblast activation or active ECM remodelling 32, 36, 267. High-resolution CT, particularly when combined with quantitative or radiomic analysis, provides an orthogonal measure of structural disease burden by capturing features such as traction bronchiectasis, honeycombing and regional tissue heterogeneity 12, 268, 269. These biomarker classes therefore interrogate different dimensions of fibrosis: immune activity, stromal activation and established structural remodelling.

In principle, integrating such information could improve therapeutic stratification. Patients with evidence of active macrophage-associated programmes might be considered for immune-modulatory or macrophage-directed strategies, whereas strong fibroblast or matrix-remodelling signatures could support evaluation of therapies targeting stromal activation, mechanotransduction or ECM turnover. By contrast, extensive honeycombing and advanced architectural distortion may indicate that a substantial component of disease is structurally established and less amenable to reversal, increasing the importance of therapies that slow further progression together with supportive management and, where appropriate, transplant assessment. These examples should currently be viewed as a conceptual framework rather than established clinical decision rules: most proposed biomarkers have not yet been prospectively validated as predictive markers for therapeutic selection.

The distinction between prognostic, pharmacodynamic and predictive biomarkers will therefore be critical. A biomarker associated with disease severity or progression does not necessarily identify the pathway that is causally maintaining fibrosis, nor does it establish that patients with high biomarker levels will preferentially respond to inhibition of that pathway. For example, circulating SPP1 may identify a macrophage-rich disease state without necessarily predicting response to SPP1-directed therapy. Similarly, POSTN or CTHRC1-associated signatures may indicate active stromal remodelling but require prospective treatment-interaction studies before they can guide selection of fibroblast- or matrix-directed interventions. Biomarker development should therefore proceed in parallel with mechanism-based therapeutic trials rather than being inferred solely from cross-sectional disease associations.

Dynamic phenotyping may further improve treatment selection because the dominant pathological programme is unlikely to remain fixed throughout disease progression. Longitudinal integration of circulating biomarkers, tissue molecular signatures and quantitative imaging could help determine whether an individual patient is transitioning from an inflammatory or injury-responsive state towards fibroblast-dominant, matrix-stabilized or immune-excluded disease. Single-cell transcriptomics, spatial profiling and multiplex imaging can provide mechanistic resolution in research settings, whereas clinically scalable blood-based biomarkers and imaging modalities will be required for repeated disease monitoring. AI-assisted image analysis may further enable quantitative tracking of regional remodelling, provided that resulting signatures are externally validated and linked to clinically meaningful outcomes.

Ultimately, a stage-specific treatment framework may require sequential or rationally combined therapy rather than simultaneous inhibition of every profibrotic pathway. Early intervention could suppress maladaptive inflammatory amplification while preserving repair; intermediate-stage treatment could disrupt immune-fibroblast feedback circuits and pathological fibroblast-state transitions; and later-stage therapy could increasingly target matrix persistence, mechanotransduction and niche stabilization. The optimal sequence will probably differ among patients according to the dominant biological programme and the degree of structural disease already present.

This approach would shift therapeutic decision-making from static diagnostic categories towards dynamic, state-based treatment selection. Rather than a model of 'one disease, one antifibrotic', pulmonary fibrosis may ultimately require a framework closer to 'one pathological state, one rational combination', in which therapeutic choice is determined by disease stage, dominant niche biology, evidence of target activity and residual capacity for tissue repair (Figure 6).

## Conclusions and future directions

Pulmonary fibrosis is increasingly understood not as a disorder driven by a single pathogenic cell type or signalling pathway, but as a failure of multicellular tissue regulation. Immune cells and fibroblasts do not simply coexist within the fibrotic lung; through reciprocal, dynamic and spatially restricted interactions, they actively shape one another's states and functions 12, 30, 31, 145. Monocyte-derived macrophages, dysfunctional T cells, inflammatory fibroblasts, CTHRC1^+^ myofibroblasts and aberrant epithelial populations become organized into interconnected pathological niches that influence whether tissue injury resolves or progresses towards persistent fibrosis. This conceptual shift has several implications. It challenges static and binary models of immune activation, places fibroblast heterogeneity and plasticity at the centre of disease pathobiology, and provides a framework for understanding why broad anti-inflammatory strategies have frequently failed to alter the course of IPF 54. More broadly, it suggests that fibrotic disease must be resolved across four interdependent dimensions: cell state, anatomical space, disease stage and intercellular interaction.

A major priority is to determine how these pathological niches arise and evolve in human disease. Most available human IPF tissues represent established or advanced fibrosis, leaving the initiating events and temporal ordering of niche formation largely inferred from experimental models rather than directly observed in patients 12, 145, 270. Longitudinal and stage-resolved clinical frameworks will therefore be essential. Integration of clinically indicated transbronchial cryobiopsy, explanted and donor lung tissue, prospective biobanks and longitudinal cohorts with serial imaging, pulmonary-function measurements and circulating biomarkers could help reconstruct the trajectory from productive repair to persistent fibrosis and identify windows during which pathological programmes remain reversible. In parallel, experimental systems should be refined to better capture ageing, repeated injury, chronic epithelial dysfunction and prolonged matrix remodelling rather than acute inflammatory injury alone.

Substantial methodological challenges must also be addressed before pathological niche biology can be translated into robust clinical tools. Current single-cell and spatial studies remain dominated by cross-sectional sampling, limiting direct inference about niche initiation, maturation, reversibility and temporal evolution 31, 145, 271, 272. Analytical pipelines, neighbourhood definitions and criteria for assigning biological meaning to spatial domains also vary across platforms and studies. Differences between human disease and experimental models in cellular composition, spatial architecture and disease kinetics further complicate mechanistic extrapolation 31. Progress will therefore require standardized and transparent analytical frameworks, reproducible definitions of spatial biomarkers and pathological niches, multicentre validation cohorts and orthogonal experimental approaches that distinguish spatial association from functional interaction. The objective should not be a single universal niche taxonomy, but a set of biologically grounded and reproducible criteria that can be compared across datasets, platforms and disease stages.

These advances could enable a transition from conventional disease classification towards niche-informed precision medicine. Current clinical assessment relies predominantly on symptoms, pulmonary function, imaging and conventional clinical variables and therefore only indirectly captures the cellular and molecular heterogeneity of fibrotic tissue 273, 274. Combining circulating biomarkers and quantitative imaging with tissue-resolved single-cell, spatial and multiplex profiling could define dominant pathological programmes, including macrophage-associated inflammation, fibroblast activation, adaptive immune remodelling and matrix-dominant mechanopathology. Artificial-intelligence-assisted analysis may facilitate integration of these multidimensional data, but its clinical value will depend on external validation, interpretability and demonstration that derived signatures improve clinically meaningful decisions. Importantly, biomarkers used for niche-guided therapy will need to move beyond prognostic association and demonstrate predictive value for treatment selection.

A niche-based framework also argues for therapeutic strategies matched to disease stage and biological state. During early or incompletely resolved injury, intervention may need to restore epithelial regeneration and constrain maladaptive inflammation without disrupting protective immune functions. As pathological immune-fibroblast circuits become established, therapeutic emphasis may shift towards interrupting reciprocal macrophage-fibroblast and lymphocyte-stromal signalling. Once fibrosis becomes strongly reinforced by persistent myofibroblast states, ECM crosslinking and mechanotransduction, combination approaches targeting stromal persistence and matrix mechanics may become increasingly important. Components of these pathways are already being explored therapeutically, but most clinical studies continue to rely on conventional enrolment criteria and endpoints rather than direct measures of pathological niche activity 7, 275-277. Incorporating mechanistic biomarkers, longitudinal molecular phenotyping and, where feasible, spatially informed patient stratification into clinical trials will be necessary to determine whether niche-directed therapies can outperform conventional stage-agnostic approaches.

Within this framework, spatiotemporal locking describes a tissue-level state rather than a new cell type, molecular pathway or discrete molecular threshold. It arises when pathological epithelial, immune and fibroblast states persist long enough to become reproducibly organized within local tissue architecture and are subsequently stabilized by reciprocal signalling, ECM remodelling, mechanotransduction and cellular memory. The transition from a reversible repair niche to a stabilized fibrotic niche is therefore not defined by the magnitude of any single signal, but by the progressive loss of coordinated restorative capacity. A reversible niche retains the capacity for inflammatory resolution, epithelial maturation, normalization of immune states, fibroblast deactivation and matrix turnover; a locked fibrotic niche is characterized by persistent disease-associated cell states, reinforced spatial neighbourhoods and matrix-dependent feedback that resist restoration of homeostasis. Criteria distinguishing these states are summarized in Table 6.

This concept generates several experimentally testable questions. Which cellular interactions are required for niche initiation, and which become dispensable once matrix-based feedback is established? Which pathological states remain reversible, and at what stage do epigenetic or mechanical memory constrain their return to homeostasis? Are apparently similar fibrotic niches conserved across patients and fibrosing interstitial lung diseases, or do distinct niche architectures require different therapeutic combinations? Addressing these questions will require integration of single-cell and spatial multi-omics with longitudinal sampling, lineage tracing, perturbational experiments and physiologically relevant human models. Such approaches should allow spatial association, temporal ordering and causal dependence to be progressively disentangled.

Ultimately, a spatiotemporal view of immune-fibroblast communication reframes pulmonary fibrosis as an ecosystem-level failure of tissue repair. Disease progression reflects not simply the persistence of activated cells, but the stabilization of pathological relationships among epithelial, immune and stromal populations within a remodelled extracellular environment. Integrating cellular state, anatomical organization, disease stage and intercellular communication may therefore enable more precise patient stratification and rational combination therapy. The longer-term objective is not merely to suppress further matrix accumulation, but to identify when pathological niches remain destabilizable and, where biological plasticity persists, to restore the coordinated multicellular programmes required for productive tissue repair.

## Figures and Tables

**Figure 1:**
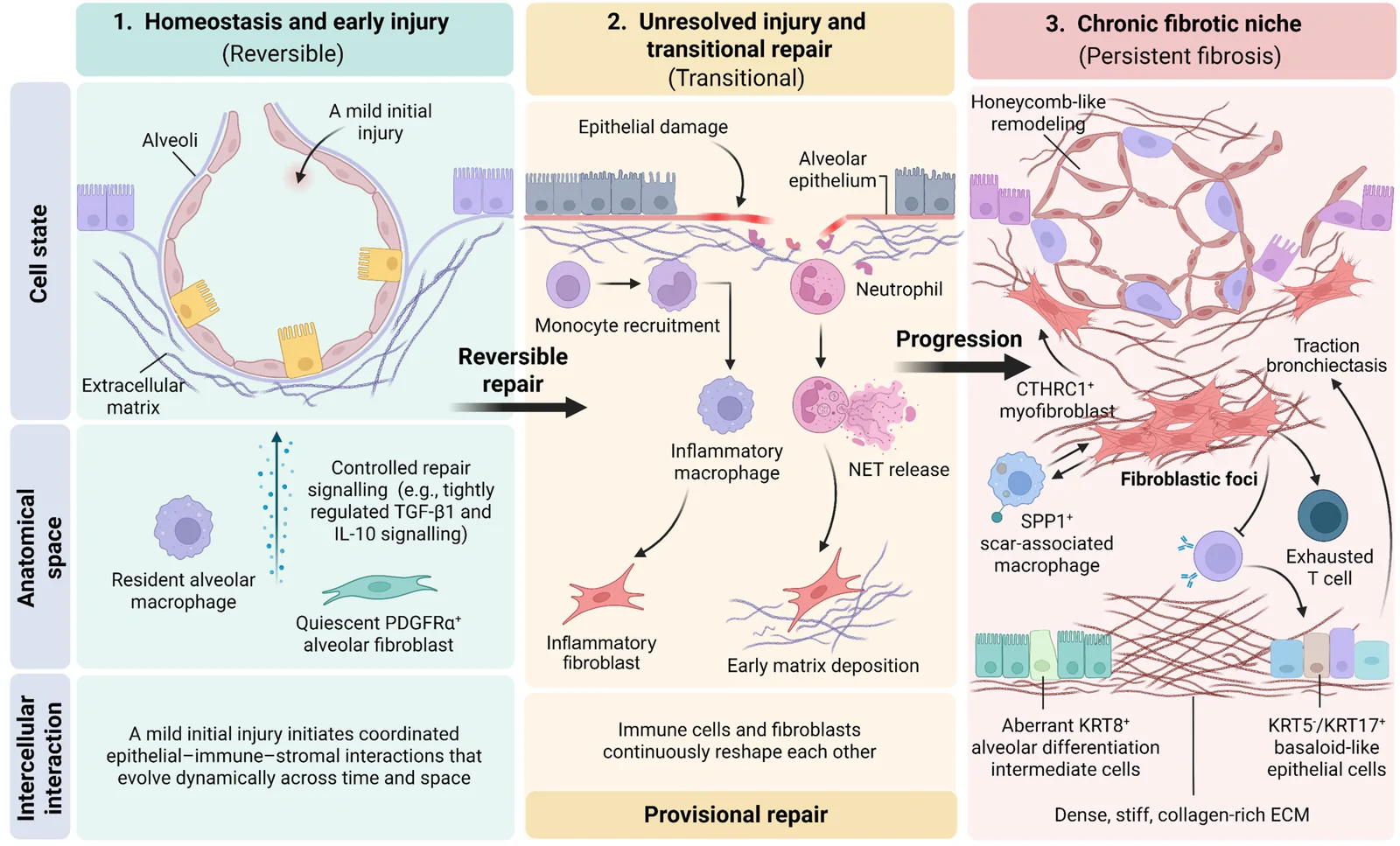
** Spatiotemporal locking of epithelial, immune and stromal circuits drives progressive pulmonary fibrosis.** Pulmonary fibrosis emerges through the progressive stabilization of pathological epithelial-immune-stromal interactions. In homeostasis and during early injury, resident alveolar macrophages, quiescent PDGFRα^+^ alveolar fibroblasts and a compliant ECM support tissue integrity and coordinated, potentially reversible repair. Persistent epithelial injury disrupts this balance and drives a transitional inflammatory state characterized by monocyte recruitment, inflammatory macrophage differentiation, neutrophil infiltration and NET formation, together with induction of inflammatory fibroblast states and early matrix deposition. At this stage, immune, epithelial and stromal populations remain functionally plastic, and their reciprocal interactions form a provisional repair niche that can still return towards homeostasis if injury resolves. When injury persists and resolution fails, however, these transient programmes progressively stabilize within chronic fibrotic niches enriched for SPP1^+^ scar-associated macrophages, CTHRC1^+^ myofibroblasts, dysfunctional or exhaustion-like T cells, KRT8^+^ alveolar differentiation intermediates and KRT5^-^/KRT17^+^ basaloid-like epithelial cells. Concurrent ECM accumulation, collagen crosslinking and matrix stiffening reinforce mechanotransductive and profibrotic signalling, while fibroblastic foci, traction bronchiectasis and honeycomb remodelling reflect increasing architectural distortion. We refer to the convergence of persistent pathological cell states, reproducible spatial organization and self-reinforcing cellular-matrix feedback as spatiotemporal locking. This process progressively reduces tissue plasticity and stabilizes fibrotic niches, converting an initially reversible repair response into a persistent, self-maintaining pathological ecosystem.

**Figure 2:**
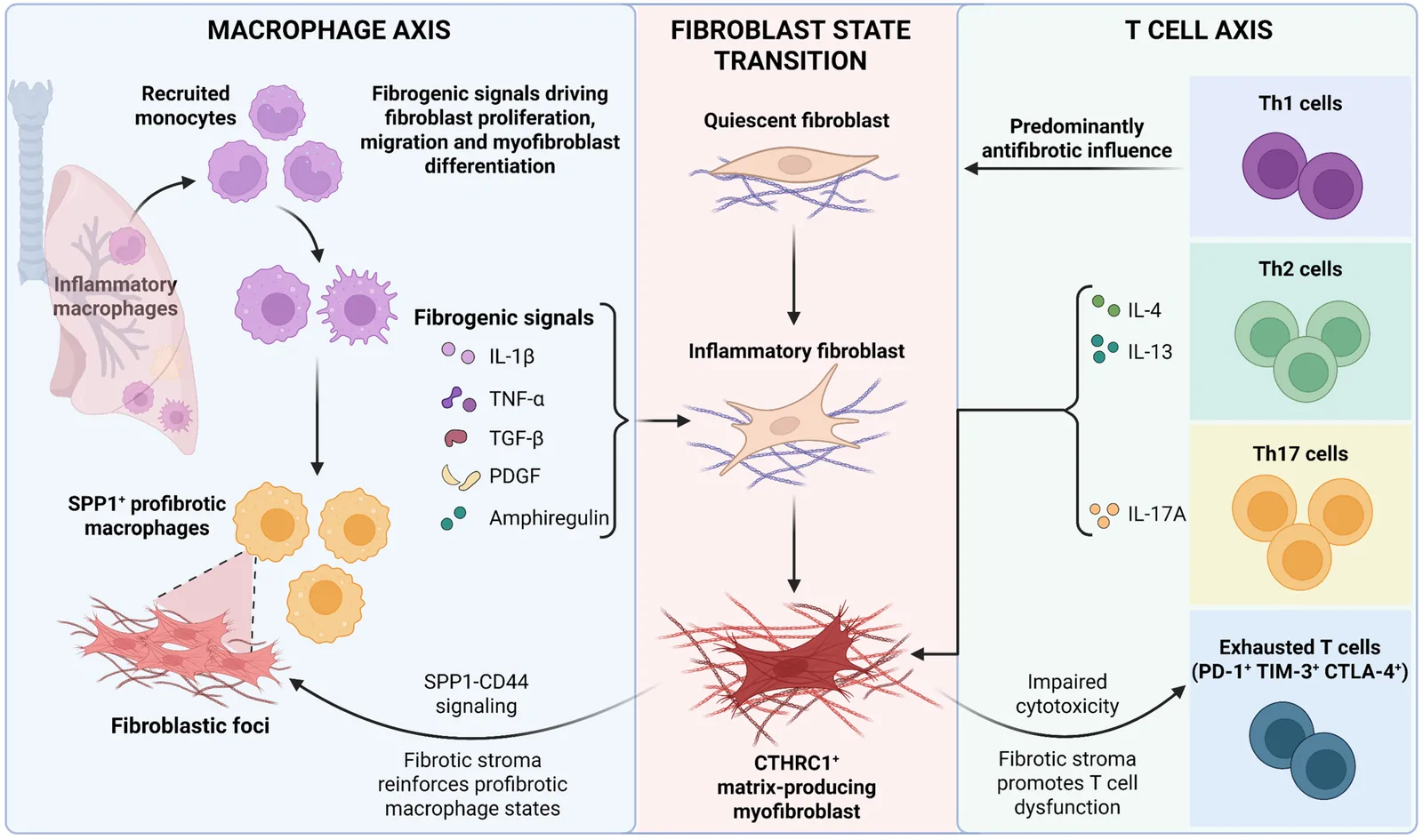
** Macrophage and T cell programmes license pathogenic fibroblast state transitions.** Innate and adaptive immune programmes converge to regulate fibroblast plasticity in pulmonary fibrosis. Following injury, recruited monocytes differentiate into inflammatory macrophages and, under conditions of persistent damage, further acquire profibrotic SPP1^+^ macrophage states. These macrophages produce mediators including IL-1β, TNF, TGF-β, PDGF and amphiregulin, thereby promoting fibroblast proliferation, migration, inflammatory activation and myofibroblast differentiation. Within fibroblastic niches, SPP1-CD44 signalling represents one candidate mechanism linking profibrotic macrophages to activated stromal cells, while fibroblast-derived signals and matrix remodelling reciprocally reinforce macrophage specialization. Adaptive immune programmes provide a second layer of regulation. Th1-associated responses generally restrain fibrosis, whereas Th2-derived IL-4 and IL-13 and Th17-derived IL-17A promote fibroblast activation and extracellular matrix production. In advanced fibrosis, progressive stromal remodelling can in turn drive T cell dysfunction or exhaustion-like states, characterized by impaired cytotoxicity and increased expression of inhibitory receptors such as PD-1, TIM-3 and CTLA-4. This loss of effective immune surveillance may limit clearance of pathogenic myofibroblasts and further stabilize fibrotic lesions. Together, macrophage-fibroblast and T cell-fibroblast circuits drive the transition from quiescent fibroblasts to inflammatory fibroblast states and CTHRC1^+^ matrix-producing myofibroblasts, thereby reinforcing the fibrotic niche.

**Figure 3:**
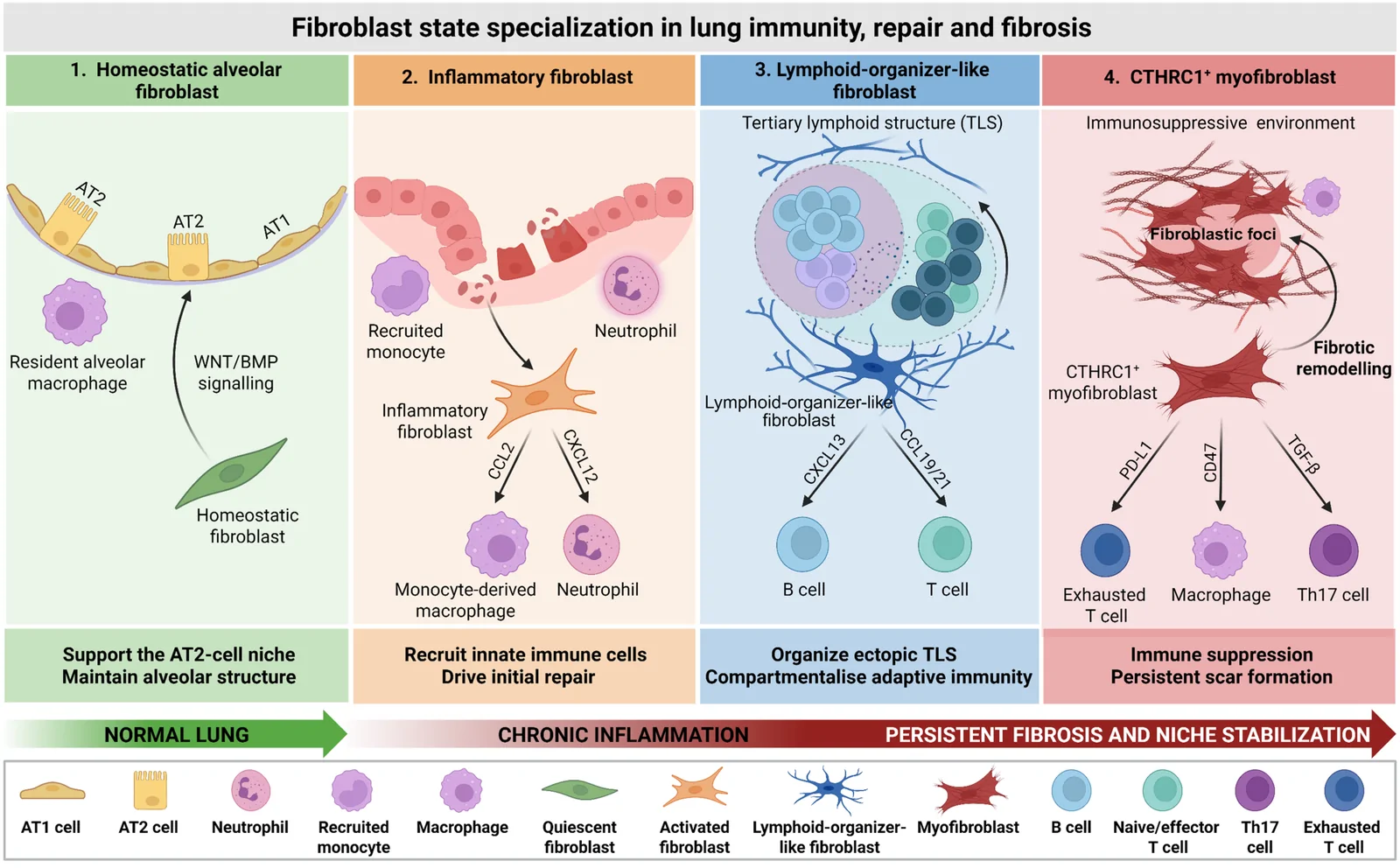
** Fibroblast state specialization links lung repair, immune organization and irreversible scarring.** Fibroblasts regulate lung repair and immunity through context-dependent state transitions. In the healthy alveolus, homeostatic alveolar fibroblasts help preserve epithelial integrity and support AT2 cell function through niche signals associated with WNT and BMP pathways. During persistent inflammation, these homeostatic programmes are progressively replaced by inflammatory fibroblast states that produce chemokines such as CCL2 and CXCL12, thereby recruiting monocytes, macrophages and neutrophils and coupling stromal activation to amplification of innate immune responses. With sustained injury, a subset of fibroblasts can further acquire lymphoid-organizer-like properties, expressing CXCL13, CCL19 and CCL21 to promote tertiary lymphoid structure formation and spatially compartmentalized adaptive immune responses. In established fibrosis, CTHRC1^+^ myofibroblasts accumulate within fibroblastic foci, deposit collagen-rich extracellular matrix and contribute to immune-suppressive scar niches through PD-L1-, CD47- and TGF-β-associated pathways. Together, these stage-specific fibroblast programmes redirect physiological alveolar repair towards chronic inflammation, ectopic lymphoid organization and persistent fibrotic scarring.

**Figure 4:**
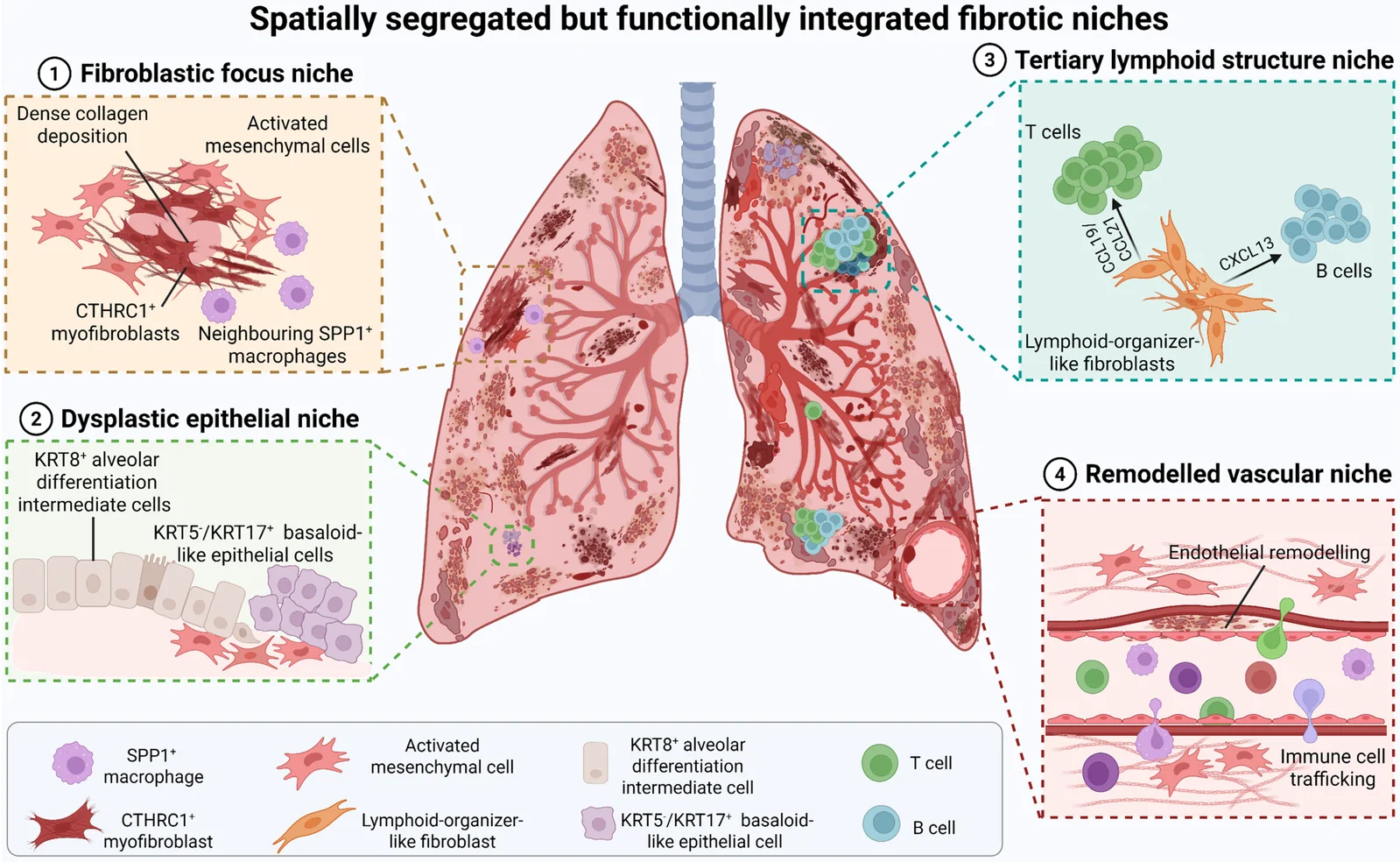
** Spatially segregated yet functionally integrated niches sustain pulmonary fibrosis.** The fibrotic lung is organized into distinct microanatomical niches that differ in cellular composition and local function yet collectively support disease persistence. Fibroblastic-focus niches are enriched for CTHRC1^+^ myofibroblasts, other activated mesenchymal populations, dense collagen-rich extracellular matrix and neighbouring SPP1^+^ macrophages, forming local hubs of matrix production and macrophage-fibroblast coupling. Aberrant epithelial niches contain KRT8^+^ alveolar differentiation intermediates and KRT5^-^/KRT17^+^ basaloid-like epithelial cells, reflecting failed alveolar regeneration and persistent epithelial stress signalling. Tertiary-lymphoid-structure-associated niches are supported by lymphoid-organizer-like fibroblasts expressing CXCL13, CCL19 and CCL21, which promote the recruitment, retention and compartmentalization of B cells and T cells. Remodelling vascular niches are characterized by endothelial dysfunction, altered immune-cell trafficking and perivascular stromal reorganization. Although these pathological niches are spatially distinct, they are functionally connected through soluble mediators, immune-cell trafficking, extracellular-matrix remodelling and mechanical feedback. Together, these local microenvironments form an interconnected spatial network that reinforces pathological cell states and sustains progressive fibrotic remodelling.

**Figure 5:**
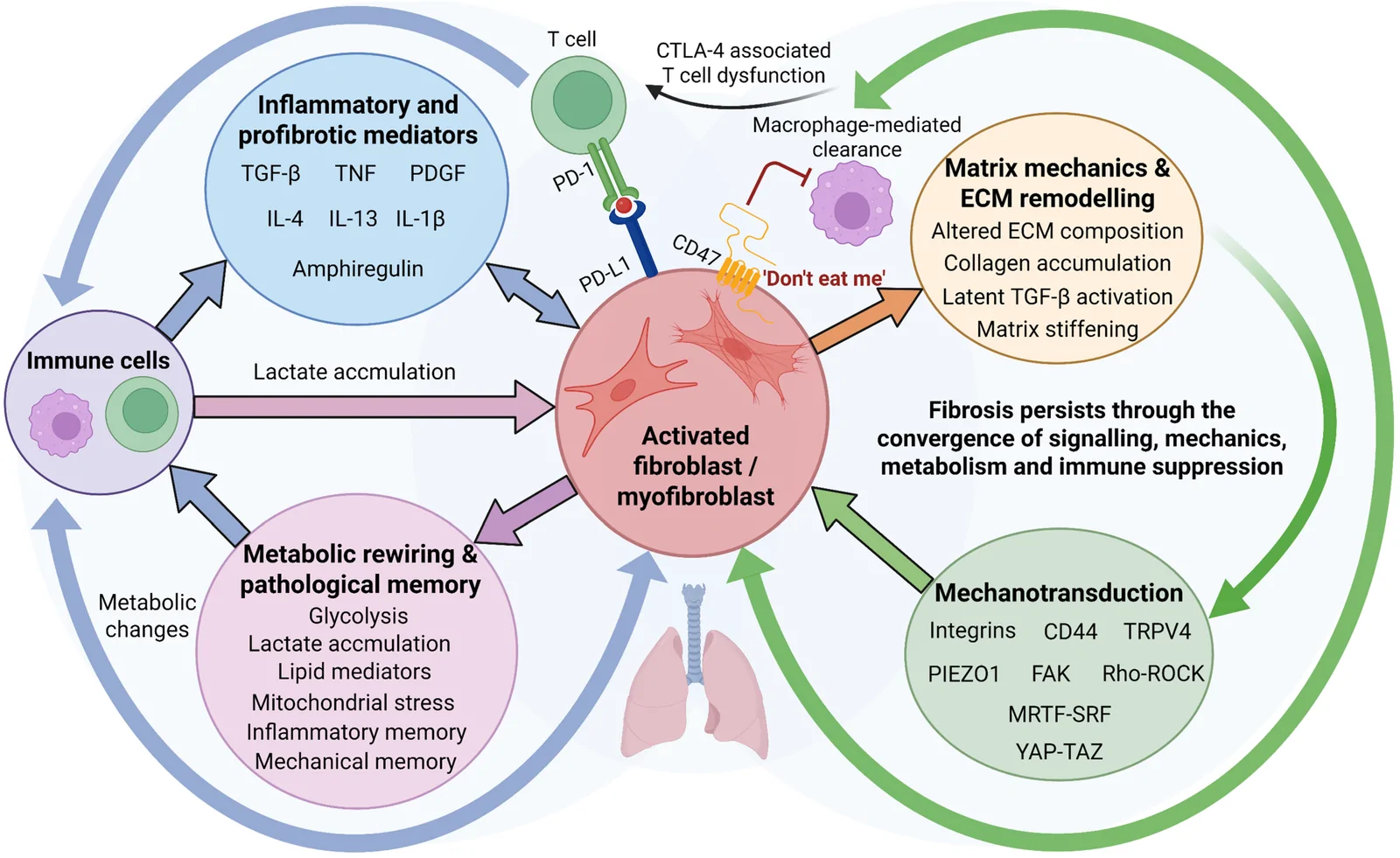
** Convergent stromal-immune circuits stabilize persistent fibrotic states.** Fibrotic persistence emerges from the convergence of soluble signalling, metabolic reprogramming, matrix mechanics and impaired immune surveillance. Activated fibroblasts and myofibroblasts integrate inflammatory and profibrotic mediators, including TGF-β, TNF, PDGF, IL-4, IL-13, IL-1β and amphiregulin, which collectively promote fibroblast activation, survival and ECM production. In parallel, metabolic reprogramming of immune and stromal cells — including altered glycolysis, lactate accumulation, lipid metabolism, mitochondrial dysfunction and redox imbalance — modifies cellular state and local intercellular signalling. Persistent metabolic, inflammatory and mechanical exposure can further establish transcriptional and epigenetic memory, reducing the capacity of pathological cell states to return towards homeostasis. Progressive ECM accumulation and collagen crosslinking increase tissue stiffness and facilitate mechanical activation of matrix-sequestered latent TGF-β. Fibroblasts sense these changes through mechanoreceptors and adhesion molecules, including integrins, CD44, TRPV4 and PIEZO1, which engage downstream FAK, Rho-ROCK, MRTF-SRF and YAP-TAZ signalling to reinforce cytoskeletal tension, myofibroblast persistence and further matrix deposition. In parallel, PD-1-PD-L1 signalling, CD47-SIRPα-mediated inhibition of phagocytosis and CTLA-4-associated T cell dysfunction can weaken immune surveillance and restrict clearance of pathological stromal cells. Together, these interconnected biochemical, metabolic, mechanical and immune-regulatory circuits reinforce one another, converting initially reversible fibroblast activation into a persistent, mechanically stabilized and locally immunosuppressive fibrotic niche.

**Figure 6:**
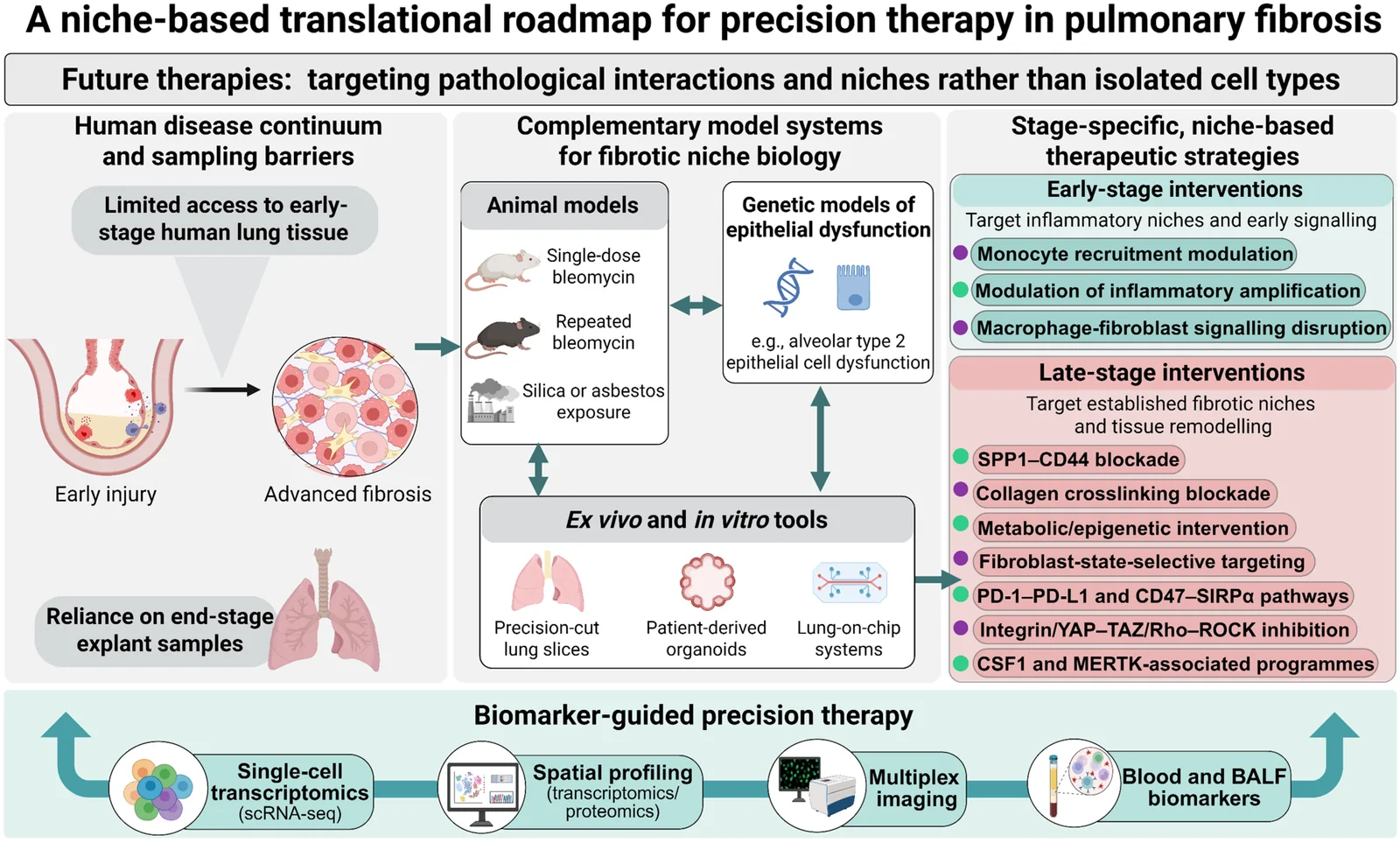
** A niche-based translational roadmap for precision therapy in pulmonary fibrosis.** Translational progress in pulmonary fibrosis requires alignment of disease stage, experimental model, biomarker profile and therapeutic mechanism. Early human disease remains difficult to sample, whereas most tissue-resolved molecular studies rely on established or advanced fibrosis, limiting direct reconstruction of the events that initiate and organize pathological niches. Complementary experimental systems are therefore required to interrogate different phases of disease: single-dose bleomycin models resolve acute injury, inflammatory recruitment and early fibrogenesis; repeated-injury, silica- and asbestos-based models better capture persistent injury and progressive matrix remodelling; genetic models interrogate susceptibility mechanisms such as epithelial dysfunction and defective regeneration; and precision-cut lung slices, patient-derived organoids and lung-on-chip systems provide human-relevant platforms for studying epithelial-immune-stromal interactions and therapeutic responses. Therapeutic strategies should likewise be matched to disease stage and dominant niche biology. During early or incompletely resolved injury, interventions may need to limit maladaptive inflammatory amplification and selected monocyte-macrophage programmes while preserving host defence and regenerative functions. As fibrotic niches become established, therapeutic emphasis may shift towards disrupting pathological macrophage-fibroblast coupling, including SPP1-CD44 and CSF1-CSF1R pathways, MERTK-associated myeloid programmes, impaired immune surveillance through PD-1-PD-L1 or CD47-SIRPα signalling, persistent fibroblast states, collagen crosslinking, metabolic and epigenetic stabilization, and integrin-, YAP-TAZ- and Rho-ROCK-dependent mechanotransduction. Longitudinal integration of circulating biomarkers and quantitative imaging with tissue-resolved single-cell transcriptomics, spatial profiling and multiplex imaging could identify dominant pathological programmes and support biomarker-guided patient stratification. Such a framework may enable sequential or rationally combined therapies to be selected according to disease stage, niche composition, target activity and residual capacity for tissue repair.

**Table 1:** Conceptual positioning of this review relative to existing frameworks

Conceptual framework	Main focus	Key advances	Remaining limitations	Contributions
Fibroblasts as immune regulators in lung fibrosis	Immunoregulatory functions of fibroblast populations	Established fibroblasts as active regulators of immune-cell recruitment, activation and resolution, rather than passive ECM-producing cells	How fibroblast-mediated immune regulation becomes spatially organized, reciprocally reinforced and chronically maintained remains incompletely defined	Extends fibroblast immunoregulation into a dynamic immune-stromal niche framework that integrates spatial organization with temporal disease progression
Innate immune networks in fibrotic interstitial lung disease	Diversity and functional states of innate immune populations	Revealed heterogeneous macrophage and other myeloid states, including fibrosis-associated macrophage programmes and complex cytokine networks	How disease-associated innate immune states interact reciprocally with fibroblast populations to establish and maintain persistent fibrotic niches remains incompletely resolved	Defines macrophage-fibroblast coupling as a reciprocal, self-reinforcing circuit rather than as parallel immune and stromal programmes
Alveolar fibroblast lineage and state transitions	Origins, trajectories and functional specialization of injury-associated fibroblast populations	Demonstrated that tissue-resident fibroblast populations undergo dynamic state transitions towards inflammatory and matrix-producing phenotypes after injury	How immune-derived signals, ECM mechanics and local spatial context jointly determine the direction, persistence and reversibility of these transitions remains unresolved	Places fibroblast-state evolution within a broader spatiotemporal regulatory landscape shaped by immune, epithelial and matrix-derived signals
Molecular pathway-centred models	Individual profibrotic mediators and signalling pathways	Identified central regulatory pathways, including TGF-β, PDGF and inflammatory cytokine signalling, and provided tractable molecular targets for antifibrotic intervention	Limited efficacy of many single-target approaches highlights pathway redundancy, compensatory signalling and strong dependence on disease stage and microenvironmental context	Reframes therapeutic targeting around stage-specific, niche-directed and rational combination strategies that disrupt interacting pathological circuits
Spatiotemporal fibrotic niche model (this Review)	Integration of cellular states, spatial organization, reciprocal communication and disease progression	Conceptualizes pulmonary fibrosis as the progressive stabilization of pathological epithelial-immune-stromal-matrix networks through reciprocal feedback and loss of tissue plasticity	Requires further longitudinal human validation, standardized criteria for niche definition and direct experimental testing of the causal mechanisms underlying niche stabilization and reversibility	Provides a unified framework linking failed repair, fibrotic niche formation, spatiotemporal locking, loss of tissue plasticity and therapeutic windows

**Table 2:** Cross-species comparison of major fibroblast and related stromal populations and their defining molecular signatures in human and murine lungs

Fibroblast or stromal population	Human defining markers	Murine defining markers	Major biological functions
Alveolar fibroblast	*NPNT, LIMCH1, TCF21*	*Npnt, Limch1, Tcf21*	l ECM homeostasis l Support of alveolar type 2 (AT2) cells l Maintenance of the alveolar epithelial niche l Regulation of alveolar repair and elastogenesis
Adventitial fibroblast	*PI16, IL33, CD34*	*Pi16, Il33, Cd34*	l ECM organization l Immune surveillance and immune-cell recruitment l Perivascular stromal support l Regulation of stromal-immune interactions
Subpleural fibroblast	*HAS1, WT1, TWIST1*	*Has1, Wt1, Twist1*	l Pleural and subpleural ECM remodeling l Hyaluronan synthesis l Maintenance of pleural mesenchymal architecture l Regulation of mesenchymal plasticity
Peribronchial fibroblast	*LGR5, COL15A1, ENTPD1*	*Lgr5, Col15a1, Entpd1*	l Airway-associated ECM organization l Structural support of the airway l Maintenance of peribronchial stromal niche l Regulation of epithelial-mesenchymal interactions
Perichondrial fibroblast	*WIF1, COL12A1, FGFR2*	*Wif1, Col12a1, Fgfr2*	l Cartilage-associated ECM organization l Modulation of WNT and FGF signaling l Maintenance of airway and cartilage-associated structural integrity
Pericyte	*PDGFRB, NOTCH3, COX4I2*	*Pdgfrb, Notch3, Cox4i2*	l Microvascular stabilization l Regulation of angiogenesis and capillary function l Maintenance of vascular integrity l Contribution to perivascular stromal signalling
CTHRC1^+^ matrix-producing fibroblast/myofibroblast	*CTHRC1, POSTN, COL1A1*	*Cthrc1, Postn, Col1a1*	l Pathological ECM deposition and collagen synthesis l Tissue contraction l Matrix remodelling and stiffening l Formation and maintenance of fibroblastic foci
Inflammatory fibroblast	*SFRP2, CCL2, CXCL12*	*Sfrp2, Ccl2, Cxcl12*	l Production of inflammatory cytokines and chemokines l Recruitment and retention of myeloid cells l Amplification of local inflammation; l Regulation of immune-stromal interactions
Immune-recruiting/lymphoid-organizer-like fibroblast	*CCL19, CCL21, CXCL13*	*Ccl19, Ccl21, Cxcl13*	l Recruitment and spatial organization of lymphocytes l Organization of stromal-immune communication l Support of tertiary lymphoid structure formation and maintenance

**Table 3:** Stage-specific immune-stromal signalling programmes driving pulmonary fibrosis and their therapeutic implications

Signalling programme	Principal cellular sources and targets	Fibrotic niche context	Most relevant disease stage	Evidence base and strength	Principal functional role	Therapeutic implications
TGF-β-SMAD signalling	Macrophages, epithelial cells and stromal cells → fibroblasts and myofibroblasts	Fibroblastic foci; matrix-remodelling and high-stiffness regions	Failed repair to established fibrosis	Strong: human IPF/fILD tissues; multiple experimental fibrosis models; extensive mechanistic studies; clinical targeting of pathway components	Fibroblast activation, myofibroblast differentiation and ECM production	Central but highly context-dependent axis; source-, stage- or activation-specific targeting may improve the therapeutic window
PDGF-PDGFR signalling	Macrophages, epithelial and stromal cells → fibroblasts	Fibroblast-expansion and remodelling niches	Early fibrogenesis to progressive fibrosis	Strong: human fibrotic lung studies; experimental perturbation; pathway targeted indirectly by approved multitarget therapies	Fibroblast proliferation, migration and survival	Clinically tractable pathway; likely to require stage-appropriate targeting rather than isolated pathway blockade
IL-1β-NF-κB signalling	Inflammatory myeloid cells and injured epithelium → immune, epithelial and stromal cells	Inflammatory failed-repair niches	Acute injury to failed repair	Moderate-strong: experimental injury models; mechanistic studies; supportive human transcriptomic evidence	Amplification of inflammation, altered stromal activation and impaired epithelial regeneration	Most relevant before structural fibrosis becomes dominant; broad suppression may interfere with host defence and repair
TNF-NF-κB signalling	Activated myeloid and lymphoid cells → epithelial cells, fibroblasts and immune cells	Immune-enriched inflammatory niches	Acute inflammation to failed repair	Moderate: human inflammatory signatures and tissue studies; extensive experimental evidence	Sustains inflammatory-stromal signalling and can modulate fibroblast activation in a context-dependent manner	Requires careful patient and disease-stage selection because TNF signalling also participates in immune defence and tissue homeostasis
IL-4/IL-13-IL-4Rα-STAT6 signalling	Th2 cells, ILC2s and other type 2 immune populations → fibroblasts and myeloid cells	Type 2 immune-stromal niches	Active fibrogenesis and failed resolution	Moderate-strong: experimental fibrosis models; human fibrotic lung and immune-profiling studies	Promotes profibrotic fibroblast programmes, collagen production and type 2 immune polarization	Potentially suited to biomarker-defined type 2-high disease contexts rather than unselected pulmonary fibrosis
IL-17A signalling	Th17 cells and other IL-17-producing lymphocytes → fibroblasts, epithelial cells and myeloid cells	T cell-stromal inflammatory niches	Failed repair to chronic fibrogenesis	Moderate: human association studies; experimental fibrosis models; mechanistic studies	Reinforces inflammatory signalling and can promote fibroblast activation and persistence	Potentially relevant to immune-phenotype-defined subsets; therapeutic window remains uncertain
SPP1-CD44/integrin signalling	SPP1^+^ disease-associated macrophages → fibroblasts and other niche-resident cells	SPP1^+^ macrophage-CTHRC1^+^ fibroblast neighbourhoods	Failed repair to progressive fibrosis	Moderate: strong human single-cell and spatial association; experimental functional support; direct human causal evidence remains limited	Reinforces macrophage-fibroblast coupling, fibroblast activation and matrix-associated niche stabilization	Attractive niche-specific target, but requires direct causal validation and strategies that preserve physiological repair
CSF1-CSF1R signalling	Stromal and other CSF1-producing cells → monocyte-derived macrophages	Macrophage-rich survival and maintenance niches	Failed repair and early establishment of fibrotic niches	Moderate: experimental fibrosis and macrophage-depletion/pharmacological studies; broader clinical validation of CSF1R targeting outside IPF	Supports survival, expansion and persistence of selected macrophage populations	Pathogenic macrophage states may need to be selectively targeted while preserving efferocytosis, host defence and reparative macrophage functions
CXCL12-CXCR4 signalling	Fibroblasts, perivascular stromal cells and endothelial cells → leukocytes and other CXCR4^+^ cells	Immune-recruitment and stromal-retention niches	Niche formation and progressive fibrosis	Moderate: human spatial/transcriptomic studies; experimental perturbation	Immune-cell recruitment, retention and spatial organization; may also influence fibroblast behaviour	Potential niche-disrupting strategy, although widespread physiological functions of CXCL12-CXCR4 constrain systemic blockade
PD-1-PD-L1 signalling	PD-L1-expressing stromal and immune cells → PD-1^+^ T cells	Locally immunoregulatory fibrotic niches	Established fibrosis	Emerging-moderate: human IPF tissue observations; experimental mechanistic studies	Contributes to T cell dysfunction and may limit immune-mediated clearance of pathological stromal cells	Exploratory strategy with a narrow safety window owing to the risk of excessive immune activation and pneumonitis
CD47-SIRPα signalling	CD47-expressing fibroblasts/myofibroblasts → SIRPα^+^ phagocytes	Fibrotic lesions with impaired stromal clearance	Established fibrosis	Emerging: human fibrotic-tissue observations and preclinical fibrosis studies	Restrains phagocytic clearance of pathological stromal cells	Potential strategy to restore stromal clearance, but systemic blockade carries substantial haematological and phagocytic toxicity
Integrin-FAK-Rho/YAP-TAZ mechanotransduction	Stiff ECM and cell-matrix adhesions → fibroblasts and other mechanosensitive cells	High-stiffness, collagen-rich fibrotic niches	Progressive matrix accumulation to established fibrosis	Strong mechanistic evidence: experimental fibrosis and human fibrotic tissue; selected pathway components have undergone clinical evaluation	Converts matrix stiffness into cytoskeletal tension, myofibroblast persistence and mechanical memory	Attractive strategy for matrix-dependent disease persistence; cell specificity and preservation of physiological mechanosensing are key
ECM crosslinking and remodelling (LOX/LOXL, MMP/TIMP)	Fibroblasts, macrophages and other stromal cells → extracellular matrix	Collagen-rich scar regions and fibroblastic foci	Progressive to established fibrosis	Strong biological, mixed translational evidence: human IPF tissue; experimental fibrosis; cross-organ studies; negative clinical experience for selected targets	Regulates matrix deposition, degradation, crosslinking, stiffness and retention/activation of matrix-bound signals	More likely to benefit from isoform-, stage- or combination-specific approaches than indiscriminate inhibition of matrix remodelling
Metabolic and epigenetic reprogramming	Macrophages, fibroblasts and epithelial cells; intracellular and intercellular metabolic coupling	Persistent pathological cell-state niches	Failed repair to established fibrosis	Emerging-moderate: human single-cell and multi-omic studies; experimental metabolic and epigenetic perturbation	Stabilizes disease-associated cell states, inflammatory/stromal memory and reduced cellular reversibility	Potentially useful for restoring cellular plasticity or sensitizing established niches to combination therapy; systemic effects remain a major limitation

**Table 4:** Clinical landscape of approved and selected investigational therapies across pulmonary fibrosis and fibrosing interstitial lung diseases

Drug	Primary mechanism of action	Key clinical evidence and outcomes	Current clinical/regulatory status	Key trials
Nintedanib	Intracellular tyrosine kinase inhibitor targeting VEGFR, FGFR and PDGFR signalling	Consistently reduced the annual rate of FVC decline in IPF; efficacy in slowing FVC decline was subsequently demonstrated in progressive fibrosing ILDs and SSc-ILD 246.	Approved	NCT01335464; NCT01335477; NCT02999178
Pirfenidone	Antifibrotic agent with incompletely defined mechanism; modulates multiple profibrotic pathways, including TGF-β-related signalling in experimental systems	Reduced decline in FVC in IPF and established efficacy as an antifibrotic therapy 247.	Approved	NCT01366209
Nerandomilast	Preferential PDE4B inhibitor with antifibrotic and immunomodulatory activity	Phase III FIBRONEER-IPF and FIBRONEER-ILD trials demonstrated significantly smaller declines in FVC than placebo at 52 weeks 7.	Approved	NCT05321069; NCT05321082
Bexotegrast	Dual αvβ6/αvβ1 integrin inhibitor designed to reduce integrin-dependent TGF-β activation and fibrotic signalling	Phase IIa INTEGRIS-IPF showed favourable short-term tolerability and exploratory signals of reduced FVC decline, imaging-defined fibrosis progression and fibrosis-associated biomarkers 257. However, the subsequent BEACON-IPF Phase IIb/III programme was terminated following an imbalance in IPF-related safety events.	Phase IIb/III programme terminated	NCT04396756; NCT06097260
Admilparant	Oral lysophosphatidic acid receptor 1 (LPA1) antagonist	In Phase II, 60 mg twice daily reduced ppFVC decline relative to placebo; the treatment difference was 1.4 percentage points in IPF and 3.2 percentage points in PPF, with the IPF confidence interval including zero 278. Phase III development is ongoing.	Phase III	NCT04308681; NCT06003426; NCT06025578
Pamrevlumab	Monoclonal antibody targeting connective tissue growth factor (CTGF/CCN2)	Despite encouraging Phase II findings, the Phase III ZEPHYRUS-1 trial did not significantly reduce FVC decline versus placebo and did not meet the primary efficacy endpoint 279.	Phase III completed; efficacy not confirmed	NCT03955146
GLPG1205	GPR84 antagonist targeting myeloid-associated inflammatory signalling	In the Phase II PINTA trial, GLPG1205 produced only a small numerical difference in FVC decline versus placebo; the study did not demonstrate a significant FVC benefit and showed poorer safety and tolerability than placebo 280.	Phase II completed	NCT03725852
Mycophenolate mofetil	Inhibition of inosine monophosphate dehydrogenase, restricting de novo guanosine synthesis and lymphocyte proliferation	In Scleroderma Lung Study II, both mycophenolate mofetil and oral cyclophosphamide were associated with improvements in FVC over 24 months; mycophenolate was not superior for the primary efficacy endpoint but was better tolerated 281.	Established immunomodulatory use; not specifically approved as an antifibrotic therapy	NCT00883129
Cyclophosphamide	Alkylating immunosuppressant that suppresses proliferating lymphocyte populations	Cyclophosphamide has demonstrated modest efficacy in selected CTD-ILD/SSc-ILD settings. Importantly, in acute exacerbation of IPF, addition of intravenous cyclophosphamide to high-dose glucocorticoids did not provide clinical benefit, arguing against extrapolation of immunosuppressive efficacy to IPF 282.	Established immunosuppressive agent in selected CTD-ILD contexts; not an IPF antifibrotic therapy	NCT02460588; NCT00883129
Rituximab	Anti-CD20 monoclonal antibody mediating B-cell depletion	In the Phase IIb RECITAL trial, rituximab was not superior to intravenous cyclophosphamide, although FVC and quality-of-life measures improved in both groups and fewer adverse events occurred with rituximab 283.	Phase IIb evidence; used in selected CTD-ILD contexts	NCT01862926
Tocilizumab	IL-6 receptor blockade	The Phase III focuSSced trial did not meet its primary skin-fibrosis endpoint, but lung-function analyses showed preservation of FVC in patients with SSc-ILD. Tocilizumab is approved in the USA to slow decline in pulmonary function in adults with SSc-ILD 284.	Approved for SSc-ILD	NCT02453256

**Table 5:** Stage-specific prioritization and translational landscape of therapeutic targets involved in immune-fibroblast crosstalk in pulmonary fibrosis

Therapeutic target	Most relevant disease stage / niche context	Evidence base	Current translational status	Near-term translational assessment	Major safety or biological concerns
SPP1-CD44	Failed repair; macrophage-fibroblast niche formation; progressive fibrosis	Human scRNA-seq and spatial studies; experimental fibrosis; mechanistic studies	Preclinical in pulmonary fibrosis	Moderate — strong niche association and biomarker potential, but direct causal and therapeutic validation in human IPF remains limited	Disruption of wound repair and immune-cell trafficking; interference with physiological stromal responses
CSF1-CSF1R	Failed repair; establishment and maintenance of macrophage-rich fibrotic niches	Human tissue studies; experimental fibrosis; pharmacological and genetic perturbation	No established IPF programme; clinically validated drug class outside IPF. CSF1R-blocking axatilimab is FDA-approved for refractory chronic GVHD	Moderate-high feasibility, moderate IPF priority — clinically tractable macrophage-directed platform, but pulmonary fibrosis-specific efficacy remains unproven	Loss of reparative macrophage functions; impaired host defence and efferocytosis; infection susceptibility
MERTK	Failed resolution; persistent disease-associated macrophage states	Human tissue association; experimental fibrosis; macrophage mechanistic studies	Preclinical in pulmonary fibrosis	Low-moderate — biologically attractive but strongly stage dependent because MERTK also supports efferocytosis and inflammatory resolution	Impaired apoptotic-cell clearance; delayed inflammatory resolution; potential exacerbation of tissue injury
CCL2-CCR2	Persistent monocyte recruitment; inflammatory-to-fibrotic transition	Human and experimental studies; lineage/depletion approaches; pharmacological inhibition	Clinical proof-of-concept attempted in IPF. Phase II anti-CCL2 therapy with carlumab failed to improve lung function or symptoms	Low-moderate — clear biological rationale, but chemokine redundancy and prior clinical failure reduce near-term priority	Impaired antimicrobial defence and reparative monocyte recruitment; compensatory chemokine pathways
PD-1-PD-L1	Adaptive immune dysfunction; failed stromal clearance; established fibrosis	Human tissue studies; experimental fibrosis; mechanistic immune studies	Early clinical evaluation in IPF. A Phase I study of the anti-PD-L1 antibody atezolizumab has been registered in IPF (NCT05515627)	Low — mechanistically provocative but narrow therapeutic window and substantial safety concerns	Immune-related pneumonitis; autoimmune toxicity; excessive inflammatory activation
CD47-SIRPα	Failed myofibroblast clearance; established fibrosis	Experimental pulmonary fibrosis; stromal immune-evasion studies; broader fibrosis evidence	Preclinical in pulmonary fibrosis; clinical platforms established mainly in oncology	Low — existing pharmacological platforms facilitate testing, but IPF-specific efficacy is unestablished and systemic toxicity is substantial	Anaemia; thrombocytopenia; non-selective phagocytosis and disruption of physiological cell clearance
αv integrins (particularly αvβ6 and αvβ1)	Active fibrogenesis; TGF-β activation; mechanically reinforced progressive fibrosis	Human tissue; strong experimental evidence; clinical trials in IPF	Clinical validation attempted. Bexotegrast showed encouraging Phase IIa signals, but the subsequent BEACON-IPF Phase IIb/III programme was discontinued in 2025 and IPF development was terminated	High biological rationale, but low-moderate near-term translational priority — target remains mechanistically compelling, but therapeutic index and target-selective delivery require reassessment	Interference with physiological TGF-β activation; impaired epithelial repair and wound healing; target- and dose-dependent toxicity
YAP-TAZ	Matrix stiffening; fibroblast-state stabilization; established fibrosis	Human fibrotic tissue; experimental fibrosis; mechanistic studies	Predominantly preclinical	Moderate biological priority, low near-term maturity — central mechanotransduction node but broad physiological roles complicate systemic targeting	Impaired epithelial regeneration and tissue repair; systemic toxicity; effects on organ homeostasis
Rho-ROCK	Active fibrogenesis; matrix-dependent fibroblast activation; established fibrosis	Experimental fibrosis; pharmacological studies; human clinical experience	Phase II IPF experience with the ROCK2 inhibitor belumosudil; broader clinical validation outside IPF. An IPF Phase II study evaluated belumosudil versus supportive care, and ROCK2 inhibition is clinically established in chronic GVHD	Moderate — druggable pathway with human pharmacology, but efficacy and optimal positioning in IPF remain uncertain	Hypotension and vascular effects with non-selective ROCK inhibition; interference with physiological cytoskeletal and repair programmes
LOX/LOXL family	ECM crosslinking; matrix stabilization; established or late fibrosis	Human tissue; experimental fibrosis; clinical targeting of LOXL2	Clinical proof-of-concept negative for LOXL2. Simtuzumab failed to demonstrate efficacy in a Phase II IPF trial	Low for LOXL2 monotherapy; potentially moderate for isoform-selective or combination approaches — matrix crosslinking remains biologically relevant despite failure of LOXL2 blockade	Impaired physiological collagen maturation and wound healing; extracellular-matrix toxicity; potential isoform compensation
Metabolic and epigenetic programmes	Failed repair through established fibrosis; pathological state persistence and cellular memory	Multi-omic human studies; experimental fibrosis; mechanistic metabolic and epigenetic studies	Predominantly preclinical and target dependent	Low-moderate — strong mechanistic rationale but heterogeneous targets and limited cell specificity; potentially attractive as combination therapy	Systemic metabolic perturbation; broad effects on normal proliferating and immune cells; cell- and pathway-specific off-target effects

**Table 6:** Proposed criteria distinguishing a reversible repair niche from a spatiotemporally locked fibrotic niche

Feature	Reversible repair niche	Spatiotemporally locked fibrotic niche
Inflammatory programme	Injury-associated inflammation resolves as the initiating stimulus is cleared	Persistent inflammatory and profibrotic programmes remain active despite attenuation of the initiating injury
Macrophage state	Macrophage populations transition towards homeostatic or inflammation-resolving states	Disease-associated macrophage states, including SPP1^+^ profibrotic populations, persist within fibrotic niches
Epithelial regeneration	AT2 cells proliferate and complete differentiation towards functional AT1 cells	Aberrant transitional epithelial states, including KRT8^+^ alveolar differentiation intermediates, accumulate and fail to complete alveolar regeneration
Fibroblast fate	Activated fibroblasts deactivate, return towards quiescence or undergo clearance after repair	Pathological fibroblast and CTHRC1^+^ myofibroblast states become stabilized and resistant to normal resolution programmes
ECM dynamics	Provisional ECM is remodelled and tissue mechanics progressively return towards homeostasis	Persistent ECM accumulation, collagen crosslinking and matrix stiffening reinforce profibrotic signalling
Cell-cell communication	Reparative epithelial-immune-stromal interactions contract as tissue integrity is restored	Reciprocal epithelial-immune-fibroblast signalling becomes self-reinforcing and sustains pathological cell states
Spatial organization	Injury-associated cellular interactions remain transient and spatially flexible	Disease-associated epithelial, immune and stromal populations form reproducible, spatially organized pathological neighbourhoods
Cellular memory and plasticity	Cellular states retain sufficient plasticity to return towards homeostatic programmes	Epigenetic, metabolic and mechanical memory constrain state reversibility and promote persistence
Dependence on initiating injury	Repair responses diminish when the initiating stimulus is removed	Fibrotic programmes become increasingly sustained by local cellular-matrix feedback rather than by the initiating injury alone
Therapeutic implication	Restoring resolution and regenerative programmes may be sufficient to recover tissue homeostasis	Effective treatment may require disruption of multiple niche-maintaining circuits together with matrix and stromal reinforcement

## Abbreviations

ALI: acute lung injury
ARDS: acute respiratory distress syndrome
AT1: alveolar type 1 epithelial cells
AT2: alveolar type 2 epithelial cells
cDC1s: type 1 conventional dendritic cells
cDC2s: type 2 conventional dendritic cells
CD44: cluster of differentiation 44
CD47: cluster of differentiation 47
CCN2: cellular communication network factor 2
CCR2: C-C motif chemokine receptor 2
CSF1: colony-stimulating factor 1
CSF1R: colony-stimulating factor 1 receptor
CTHRC1: collagen triple helix repeat containing 1
CTGF: connective tissue growth factor
CTLA-4: cytotoxic T-lymphocyte-associated protein 4
CTD-ILD: connective tissue disease-associated interstitial lung disease
CXCL12: C-X-C motif chemokine ligand 12
CXCL13: C-X-C motif chemokine ligand 13
CXCR4: C-X-C motif chemokine receptor 4
CXCR5: C-X-C motif chemokine receptor 5
DAMPs: damage-associated molecular patterns
DCs: dendritic cells
ECM: extracellular matrix
FAK: focal adhesion kinase
FDA: U.S. Food and Drug Administration
FGFR: fibroblast growth factor receptor
fILDs: fibrosing interstitial lung diseases
FVC: forced vital capacity
GPR84: G protein-coupled receptor 84
GVHD: graft-versus-host disease
HIF-1α: hypoxia-inducible factor 1 alpha
HMGB1: high mobility group box 1
IFN-γ: interferon gamma
IL-1β: interleukin-1 beta
IL-4: interleukin-4
IL-6: interleukin-6
IL-13: interleukin-13
IL-17A: interleukin-17A
IL-33: interleukin-33
ILC2s: group 2 innate lymphoid cells
ILD: interstitial lung disease
IPF: idiopathic pulmonary fibrosis
KRT8: keratin 8
LOX: lysyl oxidase
LOXL: lysyl oxidase-like
LPA1: lysophosphatidic acid receptor 1
MAPK: mitogen-activated protein kinase
MERTK: MER proto-oncogene, tyrosine kinase
MMPs: matrix metalloproteinases
mo-DCs: monocyte-derived dendritic cells
NETs: neutrophil extracellular traps
NF-κB: nuclear factor κB
NK cells: natural killer cells
PCLS: precision-cut lung slices
PD-1: programmed cell death protein 1
PDE4B: phosphodiesterase 4B
PDGF: platelet-derived growth factor
PDGFR: platelet-derived growth factor receptor
PDGFRα: platelet-derived growth factor receptor alpha
PD-L1: programmed death ligand 1
PPF: progressive pulmonary fibrosis
ppFVC: percent predicted forced vital capacity
Rho: Ras homolog
ROCK: Rho-associated coiled-coil-containing protein kinase
scRNA-seq: single-cell RNA sequencing
SIRPα: signal regulatory protein alpha
SPP1: secreted phosphoprotein 1
SSc-ILD: systemic sclerosis-associated interstitial lung disease
STAT1: signal transducer and activator of transcription 1
TAZ: transcriptional coactivator with PDZ-binding motif
TEAD: TEA domain transcription factor
TGF-β: transforming growth factor beta
TGF-β1: transforming growth factor beta 1
TIM-3: T-cell immunoglobulin and mucin domain-containing protein 3
TIMPs: tissue inhibitors of metalloproteinases
TLS: tertiary lymphoid structures
TNF: tumor necrosis factor
TREM2: triggering receptor expressed on myeloid cells 2
VEGFR: vascular endothelial growth factor receptor
YAP: yes-associated protein
